# New drugs for pharmacological extension of replicative life span in normal and progeroid cells

**DOI:** 10.1038/s41514-018-0032-4

**Published:** 2019-01-16

**Authors:** Sergei Vatolin, Tomas Radivoyevitch, Jaroslaw P. Maciejewski

**Affiliations:** 1 Department of Translational Hematology and Oncology Research, The Cleveland Clinic Foundation, Lerner Research Institute, NE6-250, 9500 Euclid Ave., Cleveland, OH 44195 USA; 2 Department of Quantitative Health Sciences, The Cleveland Clinic Foundation, Lerner Research Institute, NE6-250, 9500 Euclid Ave., Cleveland, OH 44195 USA

## Abstract

A high-throughput anti-aging drug screen was developed that simultaneously measures senescence-associated β-galactosidase activity and proliferation. Applied to replicatively pre-aged fibroblasts, this screen yielded violuric acid (VA) and 1-naphthoquinone-2-monoxime (N2N1) as its top two hits. These lead compounds extended the replicative life spans of normal and progeroid human cells in a dose-dependent manner and also extended the chronological life spans of mice and C. elegans. They are further shown here to function as redox catalysts in oxidations of NAD(P)H. They thus slow age-related declines in NAD(P)^+^/NAD(P)H ratios. VA participates in non-enzymatic electron transfers from NAD(P)H to oxidized glutathione or peroxides. N2N1 transfers electrons from NAD(P)H to cytochrome c or CoQ_10_ via NAD(P)H dehydrogenase (quinone) 1 (NQO1). Our results indicate that pharmacologic manipulation of NQO1 activity via redox catalysts may reveal mechanisms of senescence and aging.

## Remedies to extend life span

Two drugs were discovered that can extend the life spans of normally aged human cells and thus potentially slow human aging. The anti-aging drugs were identified using a novel method that screens drugs across a two-dimensional endpoint space of senescence-associated galactosidase activity as a general axis of aging and ATP as an axis representing proliferation. The two most potent substances were, likely more than coincidentally, electrons carriers that transfer electrons from NAD(P)H to molecules and cellular structures that demand reducing power to repair oxidative damage that accumulates with aging. Treatment of single cells and whole organisms with these new anti-aging drugs increased their lifespans. The mechanism of the drug action may advance our understanding of the complex, yet resolvable, biological process of aging.

## Introduction

Aging and senescence are accompanied by systemic changes in the structural integrity of cells that are caused by alterations in metabolic and signal transduction pathways.^1^ The proximal events which initiated the deterioration of complex cellular systems result in dramatic cell cycling deceleration and establishment of metabolically perplexed, irreversible non-dividing state. Indeed, early observations have indicated that normal cells are characterized by a limited replicative lifespan (RLS).^2,3^ The senesce associated β-galactosidase (SAβG) activity is considered one of the classic hall-marks of cell senescence.^4^ Among the others are telomere deterioration, multiple epigenetics changes in histones and DNA, metabolic perturbations caused by tendency of aging cells to rely more on glycolysis, hence, skewed mitochondrial dynamic toward more segregated, less respiring mitochondria.^5^ Senescence is also accompanied by increased expression of CDKN1A/2A (p21/p16) and a complex senescence-associated secretory phenotype.^6^

A small molecule high-throughput screen (HTS) requires proper selection of molecular markers for the robust and informative readout. Various approaches have been conceptualized for development of age-deceleration strategies,^7^ including, e.g., senolytics^8^ or senescence preventing strategies targeting various intracellular/extracellular pathways: telomerase machinery,^9,10^ DNA repair,^11^ nutrient response^12^ and, redox reaction.^13^ We focused on identification of agents that would prevent manifestation of classic senescence markers and overcome replicative block. We designed a new HTS for simultaneously measurement of ATP level that reflects the reenter into cell cycle and quantification of SAβG activity, an established marker of senesced cells.^4^ Our screen for anti-senescence agents generated a list of anti-aging compounds that were able to reactivate cell cycle progression in replicatively aged cells and at the same time down-regulate the SAβG activity. Direct RLS measurements in normal and progeroid human fibroblasts confirmed selection of the two most potent candidates. Detailed coverage of senescence molecular traits allowed to identify the molecular mechanisms of action of each lead compound. This work focusses on introductory characterization of anti-aging effects of violuric acid (VA) and 1-naphthoquinone-2-monoxime (N2N1) and aim to establish a potential utility of these compounds or their derivatives in prevention of cellular senescence or organismal aging.

## Results

### Screen for anti-aging agents

**Fig. 1:**
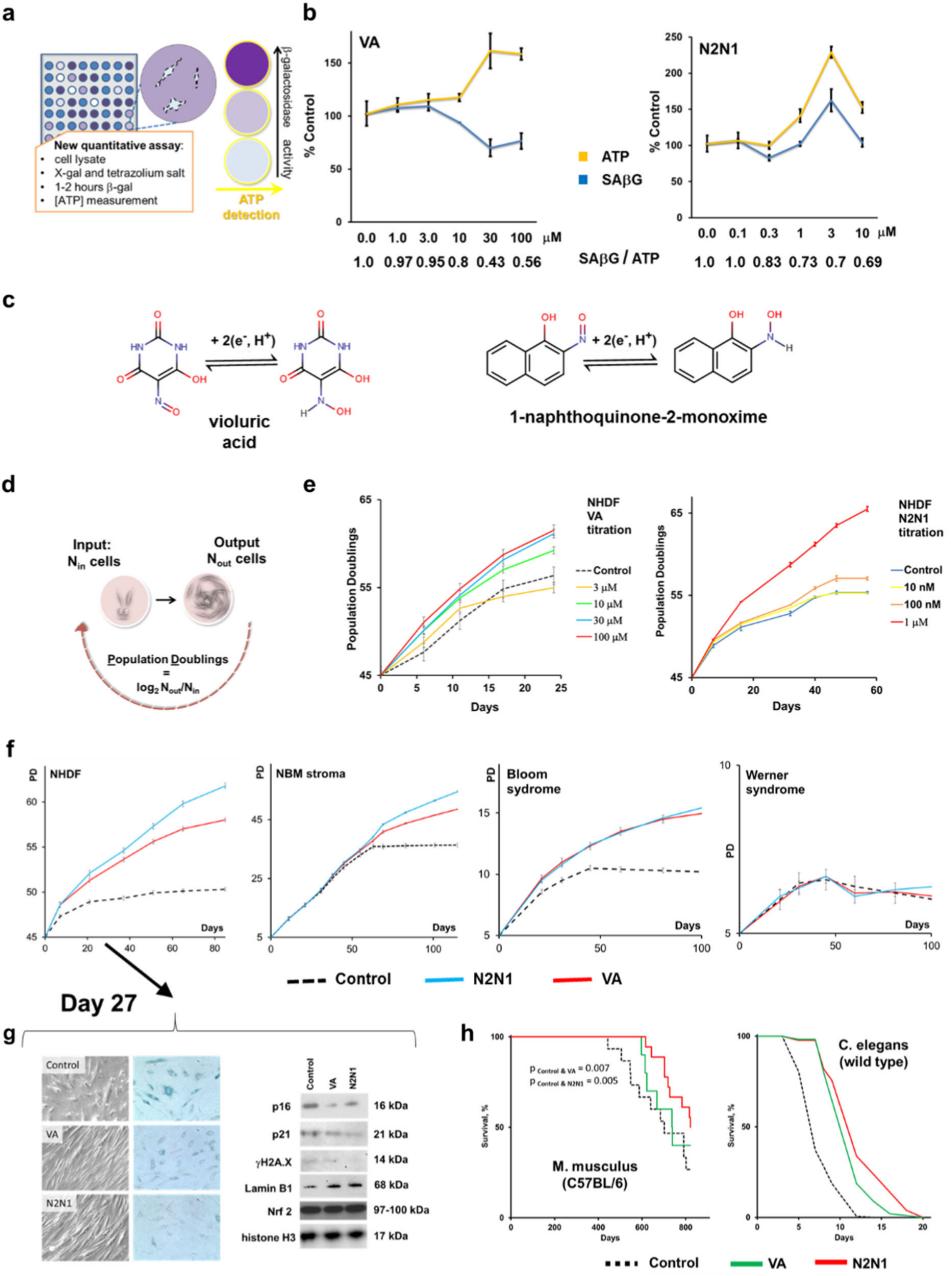
New compounds with anti-aging properties were identified using modified biochemical HTS assay for quantification of SAβG and ATP in pre-senesced cells. **a** The schema describes SAβG/ATP assay that was applied to pre-senesced NHDF. **b** Two most potent compounds, VA and N2N1, were titrated using SAβG/ATP assay. All data were normalized on untreated samples and presented as a corresponding percentile of the controls. The numbers below plots show the ratio of normalized SAβG activity divided by normalized ATP level detected in the same wells. Absolute numbers used for calculation of the ratios are shown in Table S1. Results shown are mean ± standard deviation calculated from three independent experiments (three wells per treatments in 384-well plate). **c** The chemical structures of lead compounds and possible mechanism of catalytic electrons transfer in redox reactions. **d** Schematic representation of the approach to measure the RLS in tissue culture. RLS is measured in population doublings (PD). **e** Identification of most effective concentration of VA and N2N1 in RLS extension assay applied to pre-senesced NHDF. VA was most effective at 30 μM, N2N1 at 1 μM. Results shown are mean ± standard deviation calculated from three independent experiments (three flasks per treatment). **f** VA (30 μM) or N2N1 (1 μM) were tested if they can increase RLS in normal human fibroblasts, stromal cells derived from normal bone marrow and, progeroid cells derived from the patients with Bloom or Werner syndromes. NHDF, stromal and Bloom syndrome cells responded to the treatment; Werner syndrome cells did not. Results shown are mean ± standard deviation calculated from three independent experiments (three flasks per treatment). **g** NHDF were tested after treatment with anti-aging compounds for the expression of classic markers of aging or senescence. Both VA and N2N1 decreased the expression of SAβG detected by classic histological method, down-regulated p16, p21 and γH2A.X, and up-regulated lamin B1 protein. No difference in Nrf2 protein expression was observed. All blots derived from the same experiment and were processed in parallel. **h** Increase of chronological life span (CLS) in mice or C. elegans by N2N1 or VA treatment. For the mice experiment N2N1 (100 μM) or VA (1 mM) were given in drinking water. Same concentrations of N2N1 and VA were used for the C. elegans CLS experiment. The Logrank test was used to verify the null hypothesis that there is no difference between control and treated groups (VA or N2N1). The calculated *p* values (0.007 for VA and 0.005 for N2N1) do not allow to accept the null hypothesis of no difference

The classic method for SAβG activity^4^ utilizes ferricyanide/ferrocyanide to amplify the X-gal development in formaldehyde fixed cells. The procedure requires up to 24 h and analysis with high content image examination by automated microscopy, which is time consuming and expensive. We developed a new technique (Fig. 1a) that requires considerably less time (1–2 h) to generate quantifiable signals with a very high signal-to-noise ratio. Our assay can be performed in any plate size (96-well or 384-well) that can be read by conventional plate reader. In short, β-galactosidase was released into a solution compatible with its enzymatic activity by adding Triton X100 and a catalyst, nitro blue tetrazolium salt (NBT), to shorten assay processing times. The buffer composition and formation of formazan precipitate did not interfere with ATP quantification using a standard luciferase-based approach. ATP could be detected without noticeable decay even 24 h after cell lysis and completion of SAβG activity measurements. The readout is SAβG activity (based on absorption at 615 nm) divided by normalized luciferase activity, which is proportional to ATP concentration. Such ratios were assessed relative to those of untreated control samples. Putative anti-aging drug candidates would decrease SAβG while leaving ATP unchanged or elevated; the former corresponds to normal slow-growing pre-senesced cells, the latter to growth stimulated, replicating cells. This screen thus filters outs cytotoxic compounds that decrease ATP. Compounds with lower SAβG/ATP ratios were thus considered to be better hits. We applied this approach to a small library of bioactive compounds that was augmented to include, based on our previous research, a few additional anti-aging candidates. Several compounds were selected. We focused on the top two, VA and N2N1 (Fig. 1b, c).

### Biologic properties of VA and N2N1

**Fig. 2:**
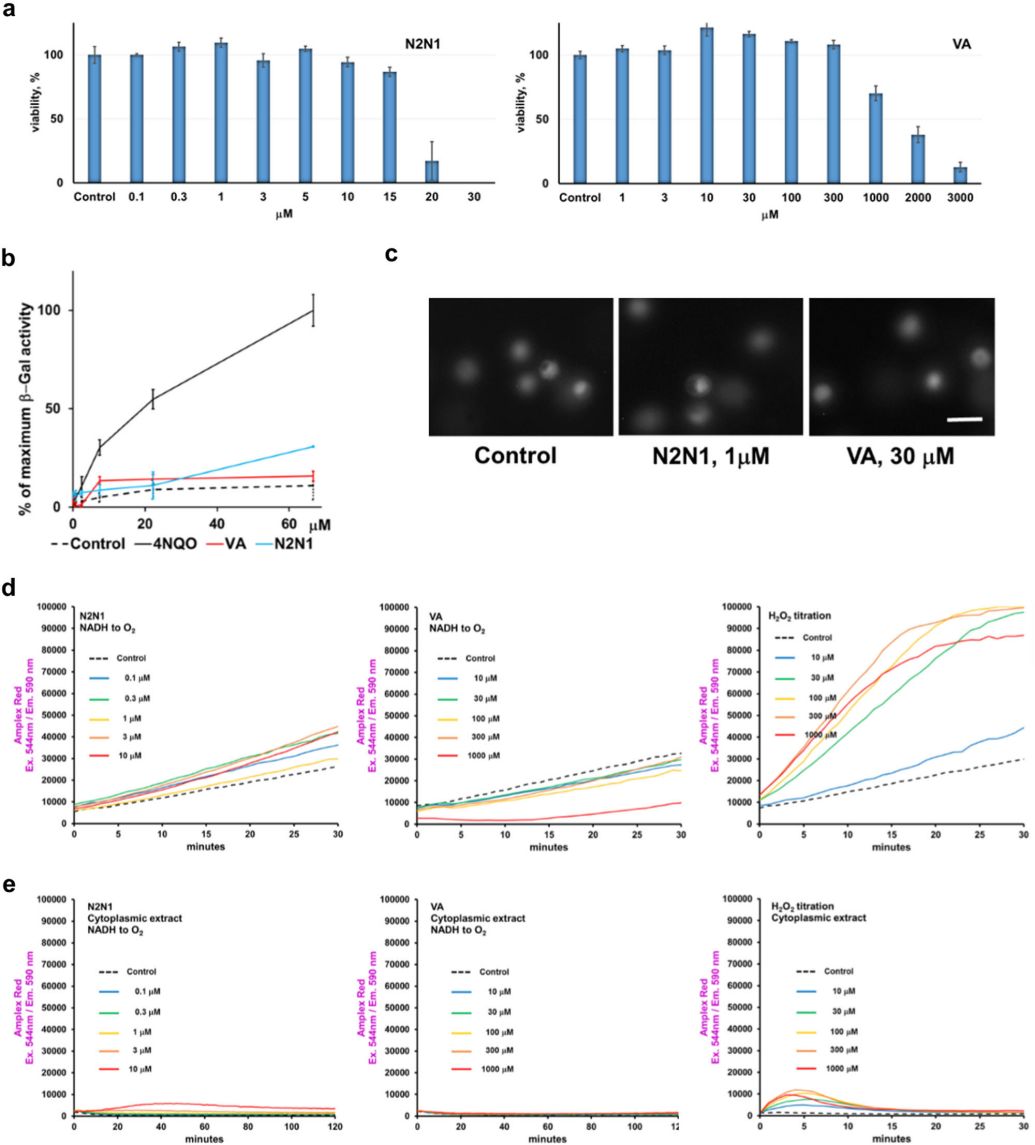
The evaluation of cellular toxicity, genotoxicity and ROS generation of N2N1 and VA. **a** The toxicity of N2N1 and VA was not observed for the concentrations used for the anti-aging studies (VA, 30 μM; N2N1, 1 μM). Much higher concentrations were tolerable by the NHDF suggesting very broad therapeutic index. Results shown are mean ± standard deviation calculated from three independent experiments (three wells per treatment in 96-well plate). **b** Genotoxic effect of VA and N2N1 were evaluated in standard Ames assay. Both VA and N2N1 did not cause any statistically significant DNA damage detectable by the assay. 4NQO (4-Nitroquinoline 1-oxide) was used as a positive control. Results shown are mean ± standard deviation calculated from three independent experiments (three wells per treatment in 96-well plate). **c** Comet assay did not show any difference between control cells and cells treated with different concentrations of N2N1 or VA. Microphotographs of control, N2N1 (1 μM) or VA (30 μM) are shown. Scale bar: 10 μm. NHDF were treated with the drugs for 10 days. Average size of the “comets” and standard deviation was calculated based on measurement of at least 25 ethidium bromide positive objects and shown in Table S2. **d** Generation of ROS was evaluated by Amplex red assay in a reaction mixture containing tested drugs and NADH. N2N1 or VA treatment did not generated any detectable level of peroxide and corresponding increase in resorufin fluorescence. VA treatment (middle plot) even slightly decreased the level of fluorescence at 590 nm indicating possible anti-oxidant properties. The H_2_O_2_ titration plot is shown on the right. **e** N2N1 or VA treatment did not result in generation of ROS in a reaction mixture containing cytoplasmic extract prepared from NHDF, tested drugs and NADH. The H_2_O_2_ titration plot is shown on the right

To further evaluate the effect of compounds on senescence phenotype in a standard tissue culture-based assay (Fig. 1d) we used several cell culture models: normal human dermal fibroblasts (NHDF), stromal/mesenchymal cells, progeroid fibroblasts derived from patients with Werner or Bloom syndromes, and a set of cancer cell lines (Fig. S1). To increase the stringency of experiments for selection of anti-aging drug candidates, NHDF were pre-aged by culturing them for up to 40 population doublings (PD): both compounds prolonged RLS in a dose-dependent manner (Fig. 1e). VA activity occurred at concentrations of 30–100 μM with no toxicity (Fig. 2a). N2N1 was most effective at 1 μM and showed some cytotoxicity above 10 μM (Fig. 2a): 10–15 additional PD were achieved with 1 μM of N2N1. VA was less effective and added 8–10 PD (Fig. 1f, left plots). Compared to untreated controls capable of 45–50 PD, VA and N2N1 increased RLS by 15–30%; in the same system rapamycin (1 nM) increased RLS by only 5–10%.^14^ VA and N2N1 restored cell cycle progression rates to those of young cells and also restored fibroblast-like morphology and decreased SAβG. When we tested N2N1 and VA on progeroid cells (Fig. 1f, right plots) derived from subjects with Bloom (BLM) or Werner (WRN) syndrome, BLM (but not WRN) fibroblasts responded to N2N1 and VA with increased proliferative capacity. BLM fibroblasts responded to N2N1 and VA by acquiring five additional PD and by increasing RLS by ~50%. VA and N2N1 down-regulated p16, p21 and γH2A.X and upregulated lamin B1 (Fig. 1g). Examined by qPCR, VA, and N2N1 affected telomere maintenance in NHDF (Fig. S2d). The culture of stromal cells derived from normal bone marrow had long telomeres at the start and at the end of the experiment. Despite this telomere maintenance, these cells experienced a broader spectrum of replicative senescence features, including p16/p21 upregulation and thus withdrawal from cell cycle (data not shown) and hypertrophic morphology. No changes in mitochondrial respiration or glycolysis were detected in the presence of the compounds (Fig. S2a, b and c). In addition, a small panel of cancer cell lines was tested with lead compounds to test the effect of drugs on cancerous phenotype (Fig. S1). We could not detect any acceleration of cell cycle progression after treatment with N2N1 or VA, probably because cultured cancer cells are already at their maximum cycling speed.

While the main focus of this study is the effect of VA and N2N1 on RLS, we also performed a pilot experiment to evaluate the possible effect of the compounds on chronological life span (CLS) in two different animal models: mouse and C. elegans. We observed an elevated survival rate during normal aging if animals received N2N1 or VA (Fig. 1h), and both compounds extended the CLS of wild-type mice and worms. To further understand the effect of VA and N2N1 on CLS, a more detailed analysis will be required with multiple aging related mutations (daf-2, age-1, clk-1 etc.), caloric load and, oxidative stress or signaling taken into consideration.

### Toxicity studies

N2N1 and VA are redox catalysts and could thus alter a wide range of biomolecules. To evaluate the cytotoxic, genotoxic, and oxidative potentials of N2N1 and VA, we performed a set of tests, results of which are shown in Fig. 2. Both compounds were tolerable, with N2N1 LD_50_ above 10 μM and VA LD_50_ above 1 mM (Fig. 2a), therefore exhibiting very broad therapeutic index beneficial for further translational research. The Ames assay and comet assay yielded that VA and N2N1 are not genotoxic (Fig. 2b, c). The Amplex red assay was used to evaluate reactive oxygen species (ROS) generation by N2N1 and VA (Fig. 2d), during direct electron transfer from NADH or electron transfer mediated by enzymes after addition to the reaction mix cytoplasmic extract prepared from NHDF (Fig. 2e). We could not detect any significant increase in resorufin fluorescence at any condition tested after treatment with VA. Addition of VA even slightly decreased the spontaneous generation of resorufin (Fig. 2d). N2N1 effect on ROS generation was vague and completely disappeared after addition of cytoplasmic extract to the reaction. While 10 μM of N2N1 increased resorufin formation slightly, this effect was quickly erased by the highly reductive capacity of cytoplasmic extract.

### VA mechanism of action

**Fig. 3:**
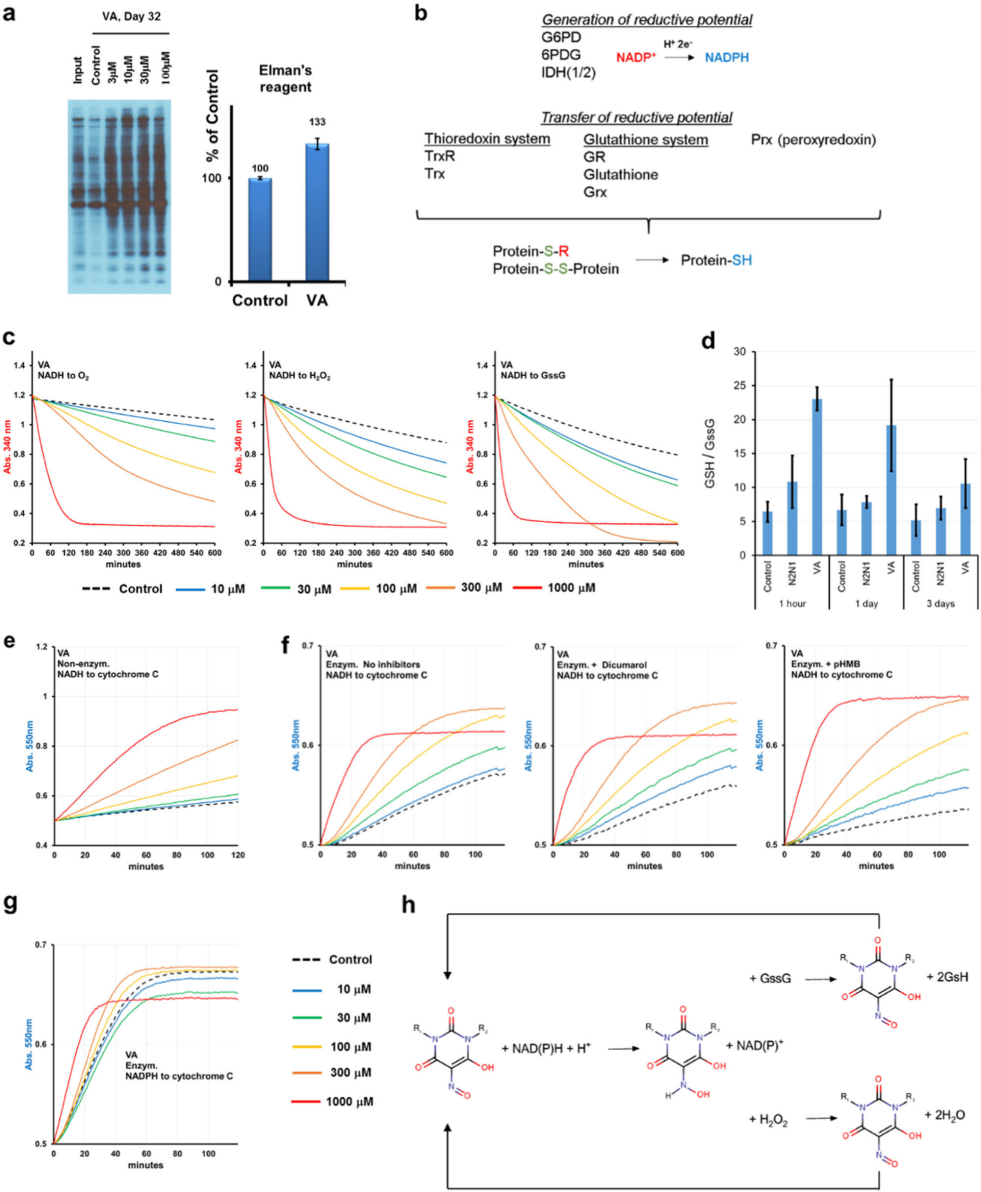
Identification and validation of VA anti-aging mechanism of action. **a** Treatment of NHDF with VA resulted in significant increase (~30%) of reduced sulfhydryl groups detected by maleimide-conjugated biotin “switch” method or by Ellman's reagent that detects reduced sulfhydryl groups to yield a colored product in solution. Results shown are mean ± standard deviation calculated from three independent experiments. **b** The redox status of proteome is under control of evolutionary conserved system that composed of enzymes that generate reductive potential in the form of NADPH and enzymes that apply electrons acquired from NADPH to the oxidized substrates. All enzymes shown on this scheme were tested with VA as a recombinant proteins or endogenous, presented in cellular or cytoplasmic extracts. The activity of none of those enzymes were modulated by VA. **c** VA can oxidize both NADH and NADPH (Fig. S4) indicating possible transfer electrons to water or molecular oxygen naturally dissolved in reaction buffer (left plot). Addition of hydrogen peroxide as a substrate increased the reaction rate (middle plot), but the most significant effect was achieved after addition of oxidized glutathione as a substrate (right plot). The reaction rates are shown in Table S3. **d** Treatment of NHDF with VA (30 μM) resulted in increase in GSH/GssG (reduced glutathione to oxidized glutathione) ratio. The effect can be observed as soon as after one hour of incubation and it can last up to 3 days. Treatment with N2N1 did show difference from control, untreated cells, but an effect did not last long. The standard deviation value is shown as an error bar and it was calculated from three independent experiments. **e** VA can transfer non-enzymatically electrons from NADH to cytochrome c. The effect can be detected only for the high concentrations of VA, 100, 300 μM and 1 mM. **f** Supplementation of the reaction mix (NADH : cytochrome c) with cytoplasmic extract did not change the overall picture. While lower VA concentrations (10 and 30 μM) were now clearly different from control, the reaction has not been inhibited neither by dicumarol nor by pHMB, the inhibitors of NQO1 and CYB5R enzymes correspondingly. Reactions were run in the presence of rotenone, 2 μM. **g** NADPH: cytochrome c electron transfer that reflects the activity of CYP (POR) enzyme family was not affected by addition of VA at any concentration tested. **h** The scheme describes the possible anti-aging mechanism of action of VA. It can oxidize both NADH and NADPH and transfer acquired electrons to either oxidized glutathione or hydrogen peroxide. The shift in GSH/GssG can cause gradual increase of reduced cysteine level in proteome and establishing more stress-resistant protein background

We found that the only persistent effect of VA treatment was considerable reduction in global protein disulfide bonds (up to 30%) detected by Elman’s reagent or direct labelling of available sulfahydril groups with a biotin-switch assay (Fig. 3a). This observation was in line with the “reductive hypothesis of aging”.^15^ We tested the enzymatic activities of thioredoxin reductase, glutathione reductase, peroxiredoxins, G6PD, 6PG, IDH1/2, LDH, MDH1/2, PDH, and αKG complexes (Fig. 3b). Reaction rates of these enzymes did not change upon treatment with VA or N2N1 (data not shown, assays protocols are in suppl. Material and methods). We then queried the possible involvement of VA in sulfhydryl oxidation. Using control samples containing all components of reaction mix except cellular extract, we detected non-enzymatic oxidation of NAD(P)H (Fig. 3c, left plot and Fig. S3a, S4a). Thus, VA likely catalyzes the non-enzymatic transfer of electrons from NAD(P)H to O_2_. In confirmatory experiments, we tested whether NADH or NADPH is a better electron donor to VA and found NADH to be oxidized at a higher rate (Fig. 3 and Fig. S3 and S4). To confirm that VA is a good lead scaffold for the development of better redox catalysts, we also tested related diphenyl- and dimethyl-violuric acid (Ox12 and Ox13; Fig. S3, S4). Whereas the diphenyl group addition increased the oxidation rate of NAD(P)H, the dimethyl group addition did not. Next, we observed that addition of GssG or H_2_O_2_ to the reaction increases the reaction rate (Fig. 3c, middle and right plots and Table S3). Both NADH and NADPH can be utilized, but NADH was oxidized faster (Fig. S3 and S4). H_2_O_2_ and oxidized glutathione were reduced better by VA, or its dimethyl analog Ox13, than by its diphenyl analog Ox12. Treatment with VA (30 μM) considerably increased the ratio of reduced to oxidized glutathione (Fig. 3d and Fig. S6b). One hour of incubation with VA was enough to push the ratio up and an effect was still observed after one day and to lesser extent after 3 days of treatment (Fig. 3d). VA also moderately increased the NAD^+^/NADH ratio after one hour of treatment and the effect may last for additional 24 h (Fig. S6a). We also tested whether VA can be used for direct electron transfer from NAD(P)H to the disulfide bonds formed inside a protein or between the proteins using a turbidity assay with insulin, but no reduction of disulfide was seen (Fig. S5).

VA could participate in non-enzymatic reduction of cytochrome c by NADH (Fig. 3e). Addition of cytoplasmic extract (with rotenone and Tx100 to inhibit mitochondrial respiration) supplemented with NADH slightly changed the reaction rates, but did not change the overall picture (Fig. 3f). Most likely, VA does not support the enzymatic electron transfer from NADH to cytochrome c. Cytochrome c can also be used as a surrogate substrate for evaluation of NADPH dependent cytochrome P450 oxidoreductases. NADPH supplemented cytoplasmic extract prepared from aged NHDF could quickly reduce cytochrome c independent of VA dose applied (Fig. 3g).

In sum, our data demonstrate that observed VA anti-aging effects on multiple cell types may stem from non-enzymatic electron transfer from NAD(P)H to H_2_O_2_ or oxidized glutathione (Fig. 3h). Impacted processes, generation of NAD^+^, cell signaling based on ROS, and intracellular redox control of glutathione, are all pivotal to aging, senescence, and stem cell maintenance.

### N2N1 mechanism of action

**Fig. 4:**
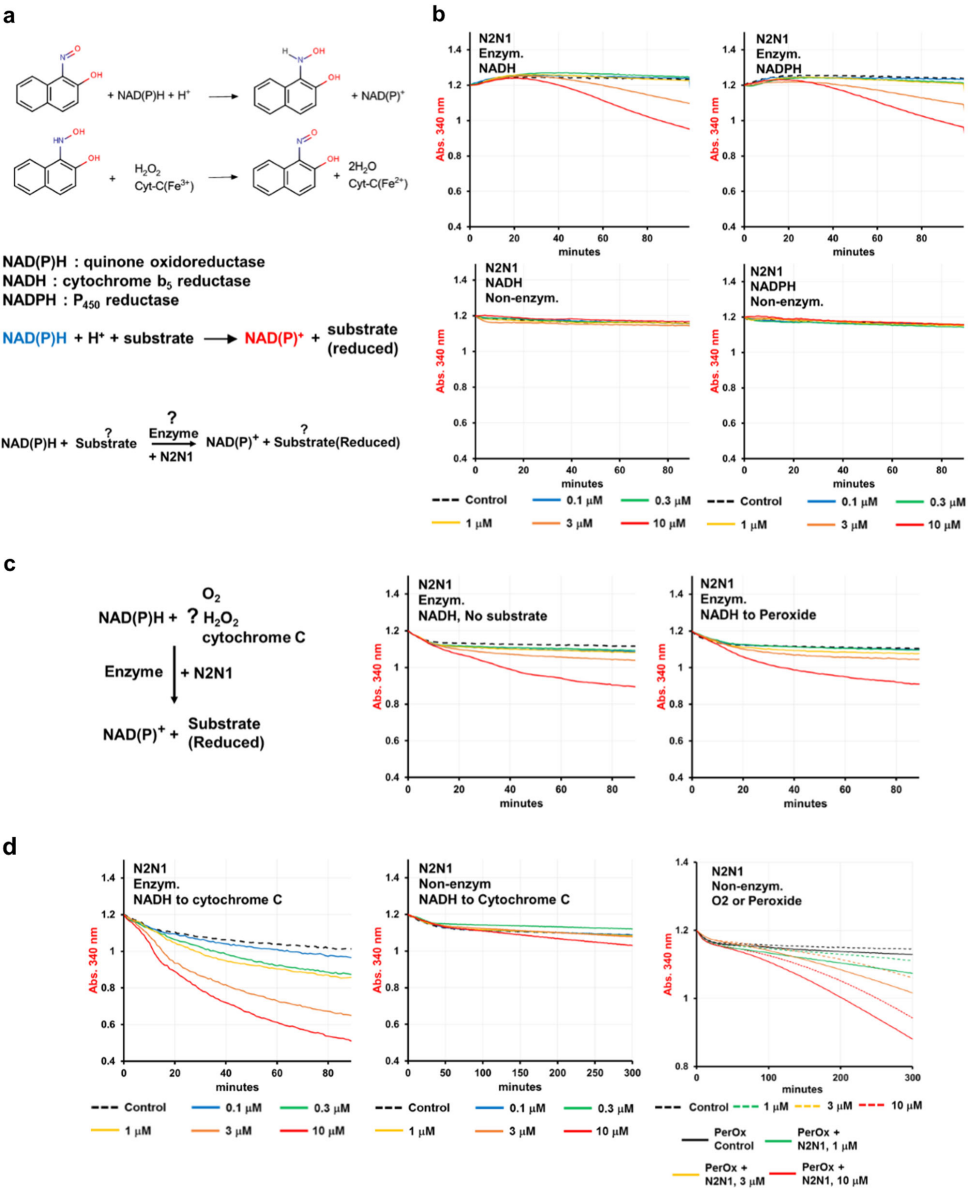
Identification and validation of N2N1 mechanism of action. **a** Previously reported biochemical properties of N2N1 include possible involvement of its isomer in redox reactions catalyzed by oxidoreductases in microsomal fractions using NAD(P)H as an electron donor. **b** N2N1 is an effective, NADH or NADPH dependent, redox mediator only in enzymatic reactions that include cytoplasmic proteins. The reaction rates are shown in Table S4. **c** Screen for the possible electron acceptor in N2N1 assisted NADH-dependent, enzymatic reactions identified cytochrome c as the preferred substrate. The reaction equation is shown on upper left plot. Addition of H_2_O_2_ did not cause a major change in the reaction rate (upper right plot); addition of cytochrome c into reaction mix increased the reaction rate from 3.4 to 26 μM/min/μg. The reaction rates are shown in Table S4. Non-enzymatic electron transfer from NADH to cytochrome c was not observed (lower middle plot, att.: 5 h of reaction are shown, compare to 90 min of enzymatic reaction). Lower left plot: Addition of peroxide into non-enzymatic reaction identified N2N1 as a week anti-oxidant (att.: 5 h of reaction are shown, compare to 90 min of enzymatic reaction). The rates for these reactions are shown in Table S4

Previous studies reported the catalytic activities of N2N1 isomers in non-enzymatic and enzymatic systems by pyridine and flavin coenzymes.^16^ These catalytic activities occurred in the presence of molecular oxygen or cytochrome c and liver-derived microsomal extracts with NADH and NADPH serving as electron donors. We therefore investigated enzymes and substrates which could partner with N2N1 as redox catalysts (Fig. 4a). N2N1 accepting electrons from NAD(P)H was confirmed by a gradual decrease in absorption at 340 nm with the addition of cytosolic enzymes into the reaction mixture (Fig. 4b, Table S4). The rate of NAD(P)H oxidation by molecular oxygen was slow (Table S4), but increased with N2N1 (at 3–10 μM, Fig. 4b). Addition of alternative substrates increased the rate of non-enzymatic NAD(P)H oxidation, while cytochrome c retarded the reaction (Fig. 4d middle plot, Table S4). Therefore, N2N1 can accept electrons from NADH and NADPH, but it prefers NADH. We compared O_2_, cytochrome c and H_2_O_2_ as electron acceptors (Fig. 4c, Table S4) in enzyme-supported reactions with N2N1. The reaction rate increased with the addition of cytochrome c, but not H_2_O_2_.

**Fig. 5:**
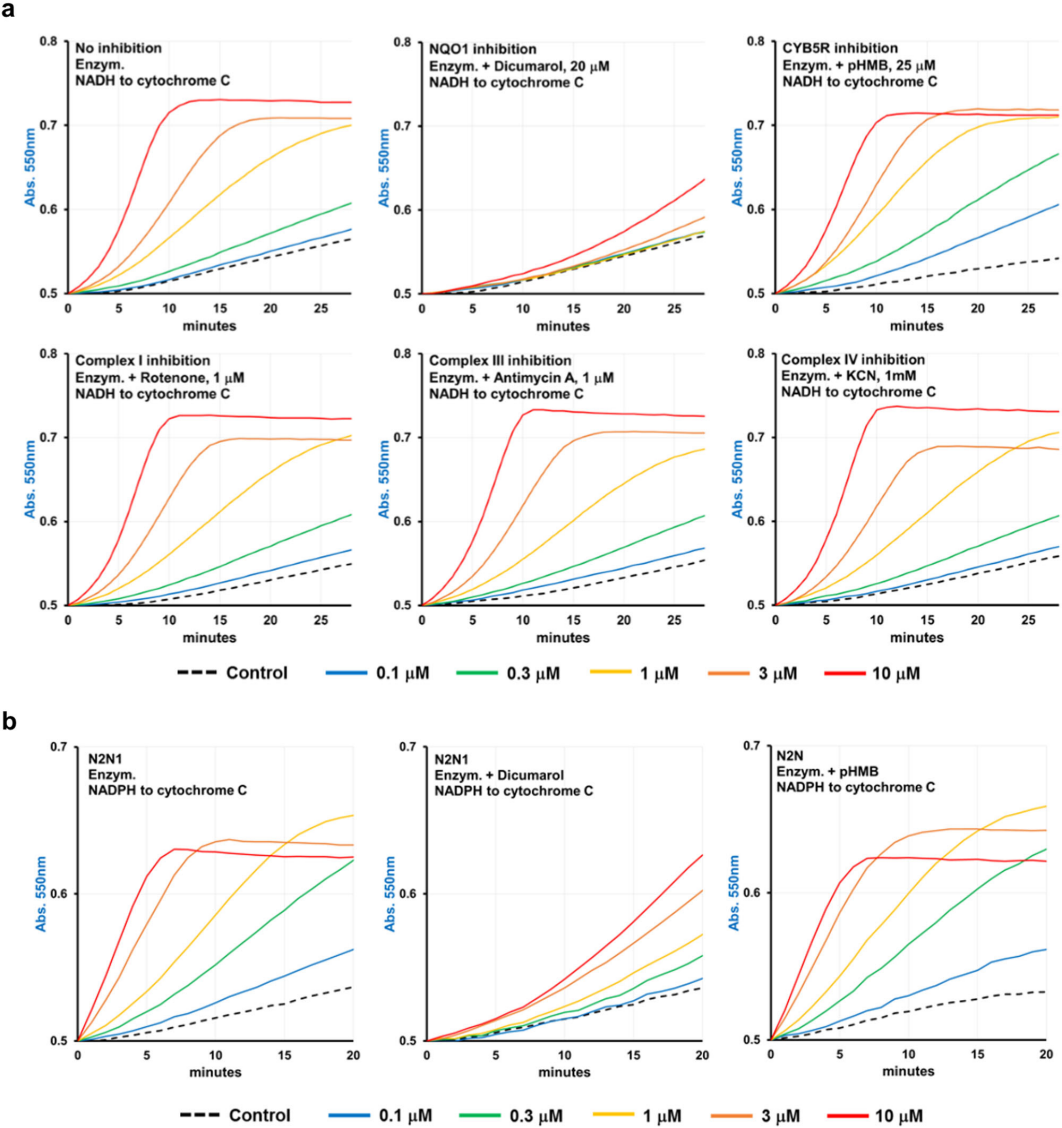
Evaluation of N2N1 assisted enzymatic reduction of cytochrome c with a set of enzyme-specific inhibitors identified NQO1 as a partner protein for N2N1-mediated anti-aging activities. Both NADH and NADPH can be the electron donors in N2N1 assisted NQO1 driven reaction. **a** Several inhibitors were added to the NADH containing reaction mix: dicumarol for NQO1, pHMB for CYB5Rs, rotenone for mitochondrial Complex I, antimycin A for mitochondrial Complex III and cyanide for mitochondrial Complex IV. Dicumarol treatment (upper middle plot) completely erased the effect of N2N1 on enzymatic electron transfer from NADH to cytochrome c. Table S5 shows the reaction rates after addition of the inhibitors and control samples. **b** The enzymatic, N2N1 assisted, NADPH-dependent reduction of cytochrome c was also inhibited only by dicumarol, pointing on NQO1 as a partner of N2N1. The residual, not-inhibited by dicumarol NADPH : cytochrome c electron transfer may indicated possible acceleration of some CYP driven enzymatic activities by N2N1. Table S6 shows the reaction rates after addition of the inhibitors and control samples

To characterize the N2N1-dependent activity, we used a set of enzyme-specific inhibitors (Fig. 5 and Table S5) with different electrons donors: NADH or NADPH. Inhibitors of mitochondrial complexes I (rotenone), III (antimycin A) and IV (cyanide) did not affect N2N1-mediated cytochrome c reduction (Fig. 5a, lower plots). Hence, we excluded the mitochondrial respiration complexes as N2N1 targets. We then determined if cytochrome c reduction could be blocked by dicumarol or pHMB, i.e., potent inhibitors of the oxidoreductases NQO1 and CYB5R, respectively. Dicumarol inhibited the reaction and pHMB did not (Fig. 5a, upper plots). Similar results were observed in NADPH: cytochrome c electron transfer: dicumarol inhibited the N2N1 assisted reaction, while pHMB did not (Fig. 5b and Table S6). It should be noted that NADPH:N2N1:cytochrome c reaction inhibited by dicumarol was not complete and less pronounced than in tests with NADH as the electron source. This raises a possibility that N2N1 may also stimulate some CYP enzyme driven reactions.

**Fig. 6:**
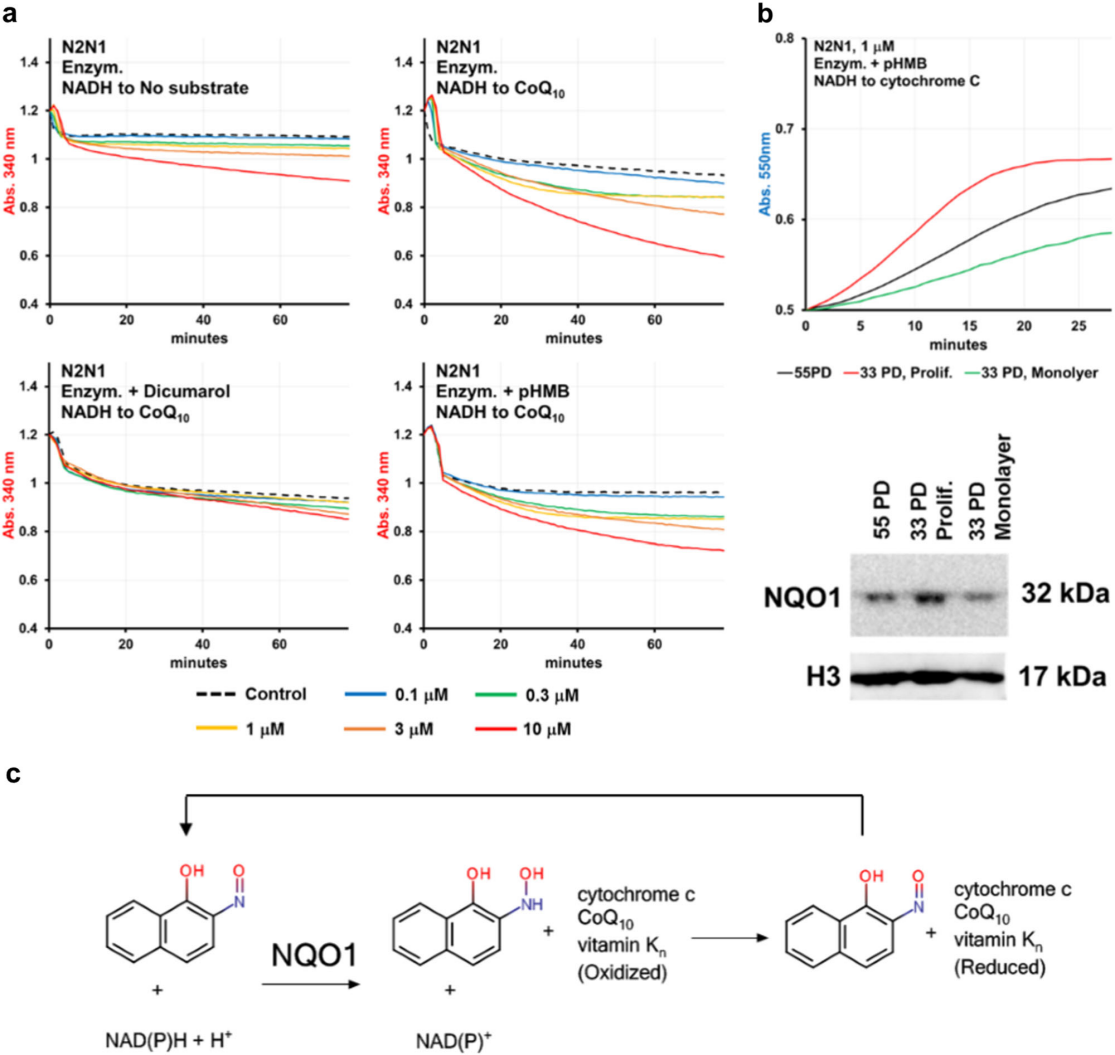
Final evaluation of N2N1 mechanism of action as a partner of NQO1. **a** CoQ_10_ can be used as a substrate in N2N1 assisted electron transfer from NADH. Reactions were supplemented with different concentrations of N2N1, pHMB or dicumarol. Rotenone was added to all reactions shown to inhibit mitochondrial Complex I activity. CoQ_10_ reduction was monitored by decreases in absorption at 340 nm (NADH oxidation). Table S7a shows the reaction rates after addition of the inhibitors and control samples. **b** Analysis of NQO1 activity and expression level in NHDF at different replicative age (55 or 33 PD) and replicative status (33 PD at active proliferation or in monolayer). Table S6b shows the reaction rates for the shown plots. Western blots were derived from the same experiment and were processed in parallel. **c** Scheme describing possible anti-aging mechanism of N2N1. N2N1 works as a redox catalyst in NQO1 mediated electron transfer from NAD(P)H to one of the enzyme’s substrate: CoQ_10_, vitamin K_n_ or cytochrome c

To elaborate more on N2N1–NQO1 mechanism, we tested the reactivity of natural NQO1 substrate CoQ_10_ (Fig. 6a and Table S7a). N2N1 catalyzed electron transfer from NADH to CoQ_10_, an effect that was inhibited by dicumarol. We also tested if well-established partners of NQO1, β-lapachone and dunnione can ameliorate the replicative decay of senesced fibroblasts. Indeed, both compounds slowed down this process (Table 1). Cell populations with NQO1 knocked out by CRISPR/Cas9 did not respond to N2N1, β-lapachone or dunnione treatments (Table 2 and Fig. S7c). Taken together, this study unequivocally identified N2N1 as a molecule whose effects depend on NQO1 enzymatic activity (Fig. 6c).

**Table 1:** The NQO1-dependent redox mediators slow down replicative deterioration of senesced fibroblasts

Treatment	μM	Day			StDv, ±^a^		
		22	30	38	22	30	38
Control		0.47	0.35	0.29	0.08	0.09	0.09
N2N1	1.0	0.57	0.47	0.43	0.11	0.08	0.09
β-lapachone	0.3	0.57	0.45	0.42	0.08	0.07	0.06
β-lapachone	1.0	0.55	0.46	0.39	0.15	0.12	0.10
(±)-dunnione	0.1	0.60	0.46	0.41	0.13	0.10	0.09
(±)-dunnione	0.3	0.57	0.40	0.41	0.14	0.10	0.10
		(PD/day)^a^					

^a^Average and standard deviation values were calculated from three independent experiments

**Table 2:** RLS in NQO1 knocked out cells cannot be augmented by NQO1-dependent redox mediator treatments

Treatment	μM	Day			StDv, ±^a^		
		7	15	24	7	15	24
Control		0.65	0.61	0.55	0.14	0.13	0.12
N2N1	1.0	0.64	0.62	0.56	0.16	0.16	0.14
β-lapachone	0.3	0.63	0.59	0.54	0.13	0.12	0.11
(±)-dunnione	0.3	0.62	0.60	0.56	0.14	0.14	0.13
		(PD/day)^a^					

^a^Average and standard deviation values were calculated from two independent experiments

## Discussion

After careful consideration of molecular markers of senescence in pre-aged cells, we decided to focus on SAβG activity and synchronous detection of ATP as a feature of cell cycle reentry. SAβG activity was measured after cell lysis in solution containing X-gal and NBT. NBT cycling substantially increases the rate of enzyme specific X-gal cleavage and thus the signal intensity. The reaction was compatible with simultaneous ATP detection via luciferase activity. This new technique was applied to normal pre-senesced human fibroblasts. It enabled selection of a lead set of small molecules with anti-aging properties.

The two most effective compounds were chosen after a secondary, confirmatory screen. They shared a molecular structure that allows them to effectively accept, carry, and donate electrons, i.e., they belong to families of redox catalysts that could potentially participate in multiple enzymatic reactions. VA is better known as a redox catalyst^17^ than N2N1.^16^ Molecules with similar structures are broadly used as redox mediators in many biochemical and medical applications,^18^ and some may contribute to the extension of RLS^13,19^ or CLS.^20,21^ Both N2N1 and VA extended RLS of NHDF and stromal cells isolated from normal bone marrow. The primary cell cultures used in our study, skin fibroblasts and especially bone marrow stromal cells, while morphologically uniform, contain a well-defined population of stem cells. For example, human skin contains a population of so-called skin-derived progenitors (SKPs).^22^ Bone marrow derived stromal culture is a very potent supplier of multipotent progenitor cells with diverse differentiation pathways.^23^ Therefore, it is possible that anti-aging drugs maintain the stem cell compartment by supporting stem cell self-renewal.

Bloom syndrome fibroblasts acquired additional replicative potential after treatment with VA or N2N1. In contrast, Werner syndrome cells were not responsive at all. Loss of BLM vs. WRN helicase may result in DNA damage repair systems that have different remaining dependencies on NAD^+^; NAD^+^ is the main substrate of poly (ADP-ribose) polymerase (PARP), which is known to be involved in alt-NHEJ, but not classical NHEJ, DNA DSB repair.

It has been reported that VA may act as an anti-oxidant^24^ or protect cells against chemically induced hypoxia.^25^ Based on our biochemical analysis we showed no influence (positive or negative) of VA on oxidative respiration, glycolysis, thioredoxin or glutathione, NADPH-generating dehydrogenases, or NADH-generating dehydrogenases (e.g., PDH or αKGDH). VA and its analogs did not modulate CYB5R and POR enzymatic activity, but VA did accept electrons from NAD(P)H and pass them along to other electron acceptors. In non-enzymatic reactions, hydrogen peroxide increased the reaction rate, indicating that VA is capable of reducing it further to water. VA was also able to donate its electrons to oxidized glutathione, thus recharging it to serve as an antioxidant. In this reaction, the rate jumped several times and that explained our original observation of elevated levels of reduced sulfhydryls after treatment of cells with VA for several days. Again, the effect of VA was quite specific for reduction of disulfide bonds in oxidized glutathione, because we could not see the reduction of disulfide bonds by VA in insulin as a substrate. Our results thus lead us to propose that VA mediates its anti-aging effect through very specific, direct reduction of oxidized glutathione and at lesser extent, reduction of peroxide. Therefore, VA and its analogs could be quite specific, with minimal side effects.

A similar approach was used to elucidate the anti-aging mechanism of N2N1. It should be emphasized that N2N1 and its isomer N1N2 exists in solution as oxime tautomers only^26,27^ and it has already been reported that molecules with a similar scaffold may have both anti-oxidant and anti-neoplastic properties.^28^ N2N1 at 1 μM increased the NAD^+^/NADH ratio after one hour of treatment and the effect became much more evident after additional 24 h (Fig. S6a). The increase in NAD^+^ level alone is an important anti-aging effect of N2N1. Indeed, pharmacologic and genetic manipulations that increase NAD^+^ levels has become a standard way to increase both chronological and replicative life spans (RLS) of cells, and is very favorable for stem cell self-renewal.^29^ Our original experimental data pointed to cytoplasmic NAD(P)H dehydrogenases NQO1, NADH-dependent cytochrome b_5_ dependent reductase (CYB5R_n_) or NADPH cytochrome P450 reductase (POR) as a possible users of N2N1. The activities of all enzymes are measured using cytochrome c as the surrogate electron acceptor from NADH or NADPH.^30^ Both NQO1^31^ and NADH-dependent cytochrome b_5_ reductase (CYB5R3) may use both NADH and NADPH and both appear to play active roles in aging.^32^ We showed that oxidative phosphorylation inhibitors did not affect the reaction and that dicumarol completely erased the effect of N2N1. We also showed that pHMB had no effect. These results allowed us to conclude that NQO1 is most likely to be the enzyme that is responsible for the N2N1 anti-aging effect. CYP enzymes could not be completely excluded from the candidate list because inhibition with dicumarol of NADPH : N2N1 : cytochrome c electron transfer was not complete (Fig. 5b).

NQO1 is a cytosolic flavoprotein-dependent enzyme that catalyzes two electron reductions of multiple substrates including quinones and vitamins K and E. NQO1 is ubiquitously expressed and can utilize NAD(P)H and some other reducing cofactors, like nicotinamide riboside.^33^ NQO1 responds to NAD(P)+/NAD(P)H balance as a redox dependent switch, promoting binding or dissociation with its target proteins: p53, p63, p73, HIF1α, centrosome localized Sirt2, and PGC1α.^34^ As a result of NQO1 elevated activity, an increase in NAD^+^ level was observed^35^ that could be beneficial for survival of replicatively or chronologically aged cells. NQO1 is considered among the most important enzymes during oxidative stress and aging due to its ability to fully reduce quinones, which pose electrophilic stress, complete reduction of semi-quinones and thus prevent oxidative damage or modify ROS-based signaling. Ubiquinol itself is a very potent scavenger of ROS of different origin and structure.^36^

The cytoplasmic extract prepared from the replicatively younger cells (33 PD) revealed more NQO1 activity (Fig. 6b and Table S7b) and slightly elevated protein expression compared to the extract prepared from the cells entering senescence crisis (55 PD) or non-proliferative young cells (33 PD, in monolyer). While there is a difference in NQO1 expression and activity between replicatively young and old cells, this variance may not be large enough to explain irreversible cell-cycle withdrawal in senescence cells. It is possible that proteins or small molecule co-factors downstream of NQO1 action are responsible for NQO1:N2N1-mediated anti-aging effects. As ROS play role both in signal transduction^37^ and as modifying agents,^38^ NQO1-dependent redox catalysts like N2N1 could be an effective anti-aging treatment by interfering with ROS-signaling. There is also a possibility that N2N1 anti-aging properties are due to NAD^+^ mediated increases in PARP activity.

NQO1 expression is under control of the transcription factor Nrf2, an important player in aging prevention and a master transcription factor of oxidative and electrophilic stress responses. Nrf2 is up-regulated in long-lived animal species.^39^ Pharmacological activation of Nrf2 pathway has been used to prevent age-related tissue dysfunctions.^40^

It has been demonstrated that a non-toxic level of NQO1-dependent redox catalyst could be an effective anti-aging treatment. β-lapachone is a NQO1 partner and its involvement in aging prevention has been well documented.^41^ In our pilot experiments (Table 1) we also demonstrated a possible anti-aging effect of β-lapachone and its derivative, dunnione. Another well-described redox catalyst,^42^ DNMA (N,N-dimethyl-4-nitrosoaniline), was inadvertently described as a potent anti-aging compound during animal treatment.^43^ As shown in Table 2 of that study, 82% of DNMA treated animals vs. 31% of controls were alive at day 600 (treatment stopped at day 420), and at day 700, 49% of treated and 0% of controls remained. The overall increase of lifespan in DNMA treated group could be 30–35% greater than that of control animals. In conclusion, we can predict that artificial redox mediators or catalysts with suitable chemical and biological properties may be good candidates for pharmacological extension of lifespan in diverse organisms.

## Methods

### Cells and tissue culture conditions

NHDF were obtained from Lonza (CC-251). Progeroid fibroblasts were purchased from the Coriell Institute for Medical Research, as were Bloom syndrome cells, (GM16891), and Werner syndrome cells (AG12798). All cells were cultured in αMEM, 10% FBS, 5 mM glutaMAX, supplemented with bFGF (StemCell technologies) and penicillin/streptomycin mix (100 U each). Stromal cells from freshly isolated normal bone marrow were isolated and propagated in MyeloCult medium, (StemCell technologies, H5100) supplemented with 1 μM of hydrocortisone. SKM-1, K562, KG-1, THP-1 cell lines were cultured in RPMI, 10% FBS, pen/strep. Growth factor dependent UT6 cells were maintained in RPMI, 10% FBS, pen/strep and erythropoietin.

### Measurement of RLS

RLS was measured in PDs (population doublings) based on formula: PD = Log_2_ (Number of cells_out_/Number of cells_input_). For the 25 cm^2^ flask, we used 10K or 30K cells per flask as an input. Cell count and viability was measured on Vi-Cell XR cell viability analyzer (Beckman Coulter).

### HTS for identification of anti-aging drugs

The screen was performed at the Case Western Reserve University School of Medicine Small Molecule Drug Development Core facility on 10K “pre-aged” (precultured for 45–50 PD) NHDF. The collection of biologically active molecules was compiled from LOPAC library (Sigma) and Bioactive Compound Library (Selleck Chemicals). A total of 2684 mechanistically annotated compounds were used for the screening. A set of redox catalysts were added to the screen, including: VA, dimethyl-nitrosoaniline, diethyl-nitrosoaniline, 1-nitroso-2-naphthol, 2-nitroso-1-naphthol, CYPMPO, DMPO, methylene blue and coniferron (Sigma-Aldrich). Stock solutions were prepared in DMSO at 10 mM. For screening, 384-well assay plates were prepared with final drug concentrations of 5 μM using a Janus liquid handling platform (Perkin Elmer) equipped with 50 nL pin transfer tool (V&P Scientific). For concentration–response studies, lead compounds were retested at eight concentrations in two-fold dilutions. A final DMSO concentration of 0.1% was not exceeded in the screening assay and in hit validation. The negative controls contained the same percentage of vehicle/DMSO. In each screening plate two vehicle columns (32 wells) served as controls. In each titration plate, four vehicle columns (64 wells) served as controls.

### Measurement of SAβG activity

SAβG activity was measured in a cell lysate after adding 50 μl of: citric acid-sodium phosphate buffer (40–60 mM, pH 6.0), 1 mM of X-gal (Gold biotechnology), 0.1 mM of nitro blue tetrazolum salt (NBT, Gold biotechnology) and 0.1% of Triton X100 (Sigma-Aldrich). After 1 h, an ATP detection solution (20 μl, Cell Titer-Glo, Promega) was added to the samples. The reagents were added by MultiFlo FX dispenser (Biotek). Sample SAβG activity was measured at 615 nm with simultaneous detection of luciferase activity as a readout of ATP using an Enspire plate reader (Perkin Elmer). Viability was calculated as a ratio of luminescence values for each compound to corresponding plate-average vehicle-only values after background subtraction. Screening data was analyzed using Pipeline Pilot (Biovia) software package. All readings were averaged and normalized on corresponding controls. The output was a ratio of SAβG activity divided by the level of ATP.

### LD_50_ evaluation

Approximately 1000 NHDF were seeded in 96-well plate (white, opaque, tissue culture treated, flat bottom, Corning) in tissue culture medium described above. After ~18–24 h, vehicle, N2N1 or VA were added at different concentrations. Cell viability was evaluated after ~96 h using Cell Titer-Glo (Promega) according to manufacturer protocol.

### Genotoxicity assay

The SOS ChromoTest kit with S9 supplement (i.e., Ames assay with liver enzyme activation) was used to evaluate potential mutagenicity of tested compounds (EBPI).

### Comet assay

Comet assay was performed according to published protocol with minor technical modifications.^44^ Briefly, treated and control NHDF were harvested and placed in 0.5–0.7% LMP agarose (at 37 °C, ThermoFisher Scientific) prepared in 1xPBS. Instead of frosted slides, to increase gel visibility and to provide a scaffold for very fragile agarose gels a simple procedure was designed. Gaskets made of filter paper (1 mm thick) were prepared with ~5 mm inside hole and 3–5 mm outside border. Gaskets were placed on a glass slide and filled with cell suspension in liquid agarose at 37 °C. Samples were placed at 4 °C until fully solidified. The resulted agarose gels can be handled with forceps and cannot be lost or damaged during lysis, electrophoresis or staining procedures. Lysing Solution: 2.5 M NaCl, 100 mM EDTA, 10 mM Tris-HCl, 0.5% Triton X100, Proteinase K ~50 μg/ml. Electrophoresis Buffer: 300 mM NaOH, 1 mM EDTA. Neutralization Buffer: 0.4 M Tris-HCl pH 7.5. Staining Solution: Ethidium Bromide ~5 µM prepared in neutralization buffer or 1×TBE.

### Amplex red assay

The reactions were set in 50 mM Tris-HCl, pH 7.5, 100 mM KCl, 2 mM MgCl_2_, 1 mM NAD(P)H, 50 μM Amplex red, with or without cytoplasmic extract prepared from NHDF. Horseradish peroxidase and Amplex red reagent were purchased from ThermoFisher Scientific. Each test (e.g., control or an experimental drug) was run in 5 independent wells, in 384-well plates (Nunc, Clear Polystyrene Plates) sealed with adhesive PCR film (ThermoFisher Scientific). All reaction mixes were prepared on ice and dispensed into cold plate (18 μl, with automatic pipette Rainin, EDP3, 0.1–1.2 ml) before addition of drug candidates (2 μl, with multichannel automatic micro-dispenser, Rainin, EDP3, 1–10 μl) or H_2_O_2_. The excitation at 544nm and emission at 590 nm were used to detect the resorufin fluorescence on Synergy H1, Hybrid reader (BioTek).

### Western blots

Cells were washed with 1xPBS and frozen at −80 °C as a pellet. Cells were lysed on ice in RIPA buffer supplemented with protease inhibitor cocktail, set V (EMD Millipore). Protein concentration was measured with Pierce BCA protein assay. Equal amounts of protein were loaded onto 4–12% polyacrylamide gel (Bio-Rad Laboratories) and electrophoresed at constant power of 45 watts, for 40 min. Proteins were transferred from gels to PVDF membranes (Bio-Rad Laboratories) per manufacturer protocols. To intensify signals, PVDF membranes after protein transfer, were air dried at room temperature and then rehydrated with 100% methanol and 1×PBS with 0.05% Tween 20. Membranes were blocked for 20–30 min at room temperature with 5% non-fat milk prepared in 1×PBS, 0.05% Tween 20. Primary and secondary antibodies (Supplementary Materials) were prepared in 5% non-fat milk, 1×PBS, 0.05% Tween-20. Peroxidase activity was measured using HyGlo (Denville scientific) detected on a ChemDoc MP Imaging system (Bio-Rad).

### GSH/GssG and NAD^+^/NADH

Glutathione measurement was measured with glutathione assay kit, Cayman chemical according to manufacturer recommendations. For NAD/NADH the protocol used was that found in the NAD/NADH quantitation kit (Biovision) used.

### Mitochondrial respiration and glycolysis

Tests were performed using Seahorse 24-well plates, XF24 sensor cartridge and a XF24 Extracellular Flux Analyzer (Seahorse Biosciences). ~50K NHDF per well. Oxygen consumption rate (OCR) and extracellular acidification rate (ECAR) were measured using the XF24 Analyzer and Assay Wizard software in replicates of 4. Drugs were added directly to the XF24 Extracellular Flux Analyzer (Seahorse Biosciences) or cells were pre-incubated with drugs for different amount of time as indicated in Fig. S2.

### Telomere length assessment

Absolute telomere length was evaluated by quantitative PCR method according to the published protocol.^45^ Cells were harvested and washed with 1×PBS. DNA was isolated using DNeasy Blood and tissue kit (Qiagen). Primers were ordered from IDT Technologies with HPLC purification provided by the manufacturer. Cycling conditions for both telomere and 36B4 products were: 40× (95 °C, 15"; 64 °C, 60") → 4 °C.

### Chronological life span

Animal experiments were performed in accordance with the Guide for the Care and Use of Laboratory Animals of the NIH, and conducted under an approved Institutional Animal Care and Use Committee protocol. CLS was evaluated in mice (C57BL/6, females) treated with vehicle (DMSO, 0.01%), N2N1 (100 μM) or VA (1 mM) in drinking water. Treatment started at the age of one month. Groups: Control, 15 animals; N2N1, 18 animals; VA, 10 animals. The daily water consumption is equal to ~3 ml. Average middle age mouse weight around 30 g would make 10 μM/kg or 1.7 mg/kg dose of N2N1 or 100 μM/kg or 17 mg/kg dose of VA.

C. elegans (wild type, N2) were obtained from Caenorhabditis Genetics Center (CGC), University of Minnesota. For CLS experiments approximately one hundred L4/young adult animals were transferred to NMG agar plates supplemented with FudR (100 μM), vehicle (DMSO, 0.01%), N2N1 (100 μM) or VA (1 mM) and kept at 25 °C. Viability was assessed every second day.

### Detection of reduced cysteines

EZ-link maleimide-PEG_2_-Biotin (ThermoFisher Scientific) procedure was used according to manufacturer protocol with ~10^6^ NHDF. Cells were lysed on ice with protease inhibitors in RIPA buffer, 200 μl. Fifty microliters of cell lysate were mixed with 500 μl of acetone and incubated at −80 °C, for 30 min. After centrifugation, at 4 °C, at maximum speed, samples were re-suspended in phosphate buffer (pH6.0) and incubated with 0.1 μM maleimide-Peg_2_-Biotin, on ice for 1 h, followed by precipitation of labelled proteins with 5 volumes of acetone. Protein concentration was measured using the BCA (bicinchoninic acid) assay. Equal amounts of protein were loaded onto 4–12% polyacrylamide gel lanes (Bio-Rad Laboratories), electrophoresed at constant power of 45 watts, for 40 min, and transferred to PVDF membranes (Bio-Rad Laboratories). As described above for Western Blots, membranes were completely dried in room temperature air, rehydrated, and blocked for 20–30 min at room temperature with 5% non-fat milk dissolved in 1×PBS with 0.05% Tween-20. To detect biotin labeled molecules, reagents A and B from Vectastain Elite ABC Kit, (Vector Laboratories) were diluted 1:1000 in 1xPBS with 0.05% Tween-20 and incubated with membranes for 1 h at room temperature.

For detection of sulfhydryl groups Elman’s reagent was used with ~10^6^ NHDF, ~40PD. Sulfhydryl groups were detected using Elman’s reagent (DTNB, 5,5′-Dithiobis(2-nitrobenzoic acid, Sigma-Aldrich). Cells were lysed on ice with protease inhibitors in RIPA buffer, 200 μl. Fifty microliters of cell lysate was mixed with 500 μl of acetone and incubated at −80 °C, for 30 min. After centrifugation, at 4 °C, at maximum speed, pellets were re-suspended in phosphate buffer (pH7.0) and protein concentrations were measured using the BCA assay. Equal amounts of protein from each analyzed sample were mixed with DTNB (0.5 mM)-GuHCl and absorbance was measured at 412 nm (Synergy H1, Hybrid reader, BioTek).

### Cytoplasmic extract preparation

Cell pellet (2–3 × 10^6^ cells, ~35–50 μl) was re-suspended in 200 μl of ice-cold CE buffer (NaCl 10 mM, MgCl_2_ 1.5 mM, Tris-HCl 10 mM, pH7.5) supplemented with protease inhibitors and incubated on ice for no more than 3 min. After that, cell suspension was centrifuged at 0.6 rcf, 4 °C, for 3 min. Supernatant was carefully transferred into a new, pre-chilled tube.

### Biochemical assays

Each test (e.g., control, an experimental drug at concentration N μM etc.) was run in five independent wells, in 384-well plates (Nunc, Clear Polystyrene Plates) sealed with adhesive PCR film (ThermoFisher Scientific). All reaction mixes were prepared on ice and dispensed into cold plate. The reaction mix (18 μl) before addition of drug candidates or corresponding substrate (2 μl) was dispensed using automatic pipette (Rainin, EDP3, 0.1–1.2 ml) with dispensing function. Experimental drugs were added with multichannel automatic micro-dispenser (Rainin, EDP3, 1–10 μl). The internal well size of 3.45 × 3.45 mm with 20 μl reaction mix volume generates the path equal to 1.6 mm. The reaction rates were calculated based on the following formulas: Δ[NAD(P)H] = ΔAbsorbance_340nm_/(6220 M^−1^ cm^−1^ × 0.168 cm); Δ[cytochrome c] = ΔAbsorbance_550nm_/(21,100 M^−1^ cm^−1^ × 0.168 cm). All experiments were performed on Synergy H1, Hybrid reader (BioTek). The measurement of absorbance at corresponding wave-length was taken every minute, at 37 °C.

#### Assays for VA

The reactions were set in 100 mM potassium phosphate buffer, pH 7.0, 1 mM NAD(P)H with 0.5 mM GssG (oxidized glutathione) or 5 mM hydrogen peroxide. The oxidation of NAD(P)H was monitored as an absorbance decrease at 340 nm. The turbidity assay with 0.1 mM of insulin was run with change of absorbance measured at 650 nm, without vortexing.

#### Assays for N2N1

The reactions were set in 50 mM Tris-HCl, pH 7.5, 100 mM KCl, 2 mM MgCl_2_, 1 mM NAD(P)H, 0.1% Triton X100 and, 0.1 mM of cytochrome c or CoQ_10_ and, freshly prepared cytoplasmic extract. Triton X100 was added into reaction mix to fully inhibit mitochondrial Complex I activity.^46^ Cytochrome c 10 mM stock solution was prepared in water. To prepare CoQ_10_ 10 mM stock solution, CoQ_10_ powder was dissolved in absolute ethanol-Triton X100 mixture (90–10%) and kept at 70 °C for 5 min. Corresponding inhibitors (dicumarol, 25 μM; pHMB, 50 μM; rotenone, 2 μM; antimycin a, 2 μM; KCN, 1 mM) were added where required. The reduction of cytochrome c was observed as an increase of absorbance at 550 nm along with oxidation of NAD(P)H monitored as an absorbance decrease at 340 nm. The reduction of CoQ_10_ into ubiquinol was evaluated based on oxidation of NAD(P)H (decrease in absorbance at 340 nm).

### CRISPR/Cas9 knockout generation

The CRISPR/Cas9 knockout plasmid with an insert to inactivate NQO1 was manufactured by Santa Cruz Biotechnology and used according to manufacturer recommendations. Primary cells, like NHDF, grow well only as relatively “dense” populations. The use of cellular populations instead of cell clones shortens times needed to detect knockout phenotypes. To generate stably knocked out cells, 10^5^ NHDF cells at ~37 PD were seeded into five 25 cm^2^ flasks. After two days, medium was changed to OPTI-MEM, 10% FBS (no antibiotics) and 10 μg of CRISPR/Cas9 plasmid was transfected into the cells using the manufacturer’s transfection reagent (Santa Cruz Biotechnology). Three days after transfection, cells were harvested from the culture flasks and sorted on a Sony SH2 800 cell sorter using a GFP expression marker. 5 GFP positive cells were collected in a single well of 96-well plate. Total 12 wells were filled with GFP positive cells. Cells were grown in αMEM, 10% FBS, bFGF, pen/strep and scaled up to at least 10^5^ cells in 25 cm^2^ flasks. The overall procedure added up to 7–10 PD. Each GFP positive population was tested for NQO1 expression by western blot (Fig. S7c). The cell population with presumably stable NQO1 knockout was used to quickly evaluate replicative capacity, measured in PD/day. N2N1 was used as a positive control, as it always speeds up cell cycling during first days of treatment, though it cannot prevent senescence, as cells continue to slow down their cell cycle, even in the presence of N2N1.

## Supplementary information

Supplemental Materials

## Data Availability

All data are available from the authors on request.

## Supplementary information

**Supplementary information** accompanies the paper on the *npj Aging and Mechanisms of Disease* website (10.1038/s41514-018-0032-4).

## References

[1] Childs BG, Durik M, Baker DJ, van Deursen JM (2015). Cellular senescence in aging and age-related disease: from mechanisms to therapy. Nat. Med..

[2] Swim HE, Parker RF (1957). Culture characteristics of human fibroblasts propagated serially. Am. J. Hyg..

[3] Hayflick L, Moorhead PS (1961). The serial cultivation of human diploid cell strains. Exp. Cell Res..

[4] Dimri GP (1995). A biomarker that identifies senescent human cells in culture and in aging skin in vivo. Proc. Natl Acad. Sci. USA.

[5] Greenberg EF, Vatolin S (2018). Symbiotic Origin of Aging. Rejuvenation research.

[6] Coppe JP, Desprez PY, Krtolica A, Campisi J (2010). The senescence-associated secretory phenotype: the dark side of tumor suppression. Annu Rev. Pathol.-Mech..

[7] Longo VD (2015). Interventions to slow aging in humans: are we ready?. Aging Cell.

[8] Kirkland JL, Tchkonia T (2015). Clinical strategies and animal models for developing senolytic agents. Exp. Gerontol..

[9] Jaskelioff M (2011). Telomerase reactivation reverses tissue degeneration in aged telomerase-deficient mice. Nature.

[10] Sahin E (2011). Telomere dysfunction induces metabolic and mitochondrial compromise. Nature.

[11] Li J (2017). AGING A conserved NAD(+) binding pocket that regulates protein-protein interactions during aging. Science.

[12] Johnson SC, Rabinovitch PS, Kaeberlein M (2013). mTOR is a key modulator of ageing and age-related disease. Nature.

[13] Xiong ZM (2017). Anti-aging potentials of methylene blue for human skin longevity. Sci. Rep..

[14] Lerner C (2013). Reduced mammalian target of rapamycin activity facilitates mitochondrial retrograde signaling and increases life span in normal human fibroblasts. Aging Cell.

[15] Jones DP (2015). Redox theory of aging. Redox Biol..

[16] Leskovac V, Svircevic J, Trivic S, Popovic M, Radulovic M (1989). Reduction of aryl-nitroso compounds by pyridine and flavin coenzymes. Int. J. Biochem..

[17] Bisogno FR, Lopez-Vidal MG, de Gonzalo G (2017). Organocatalysis and biocatalysis hand in hand: combining catalysts in one-pot procedures. Adv. Synth. Catal..

[18] Yoo EH, Lee SY (2010). Glucose biosensors: an overview of use in clinical practice. Sensors.

[19] Atamna H (2008). Methylene blue delays cellular senescence and enhances key mitochondrial biochemical pathways. Faseb J..

[20] Edamatsu R, Mori A, Packer L (1995). The spin-trap N-tert-alpha-phenyl-butylnitrone prolongs the life-span of the senescence-accelerated mouse. Biochem. Biophys. Res. Commun..

[21] Saito K, Yoshioka H, Cutler RG (1998). A spin trap, N-tert-butyl-alpha-phenylnitrone extends the life span of mice. Biosci. Biotech. Bioch.

[22] Thulabandu V, Chen D, Atit RP (2018). Dermal fibroblast in cutaneous development and healing. Wiley interdisciplinary reviews. Develop. Biol..

[23] Bara JJ, Richards RG, Alini M, Stoddart MJ (2014). Concise review: bone marrow-derived mesenchymal stem cells change phenotype following in vitro culture: implications for basic research and the clinic. Stem Cells.

[24] Burbello AT, Baskovich GA, Dobrokhotova EG, Slesarev VI (1991). Protective effect of antioxidants in methemoglobinemia caused by sodium nitrite in experimental studies. Gigiena truda i professionalanye zabolevaniia.

[25] Burbello AT, Vishvtseva VV, Denisenko PP, Safonova AF, Dobrokhotova EG (1995). The limitation of glucose catabolism as a factor in protection during hypoxia. Eksp.'naia i Klin. Farmakol..

[26] Ivanova G, Enchev V (2001). Does tautomeric equilibrium exist in ortho-nitrosonaphthols?. Chem. Phys..

[27] Krzan A, Mavri J (2002). Nitroso-naphthol quinone-monooxime tautomeric equilibrium revisited: evidence for oximo group isomerization. Chem. Phys..

[28] Zaware SB, Gonnade RG, Srinivas D, Khan A, Rane SY (2011). Antioxidant and anticancer activities of supramolecularly controlled magnetostructural halo-oximes of lawsone. New J. Chem..

[29] Rimmele P (2014). Aging-like phenotype and defective lineage specification in SIRT1-deleted hematopoietic stem and progenitor cells. Stem Cell Rep..

[30] Guengerich FP, Martin MV, Sohl CD, Cheng Q (2009). Measurement of cytochrome P450 and NADPH-cytochrome P450 reductase. Nat. Protoc..

[31] Navas P, Villalba JM, de Cabo R (2007). Importance of plasma membrane coenzyme Q in aging and stress responses. Mitochondrion.

[32] de Cabo R, Siendones E, Minor R, Navas P (2010). CYB5R3: a key player in aerobic metabolism and aging?. Aging-Us.

[33] Friedlos F, Jarman M, Davies LC, Boland MP, Knox RJ (1992). Identification of novel reduced pyridinium derivatives as synthetic co-factors for the enzyme DT diaphorase (NAD(P)H dehydrogenase (quinone), EC 1.6.99.2). Biochem. Pharmacol..

[34] Siegel D (2018). Redox modulation of NQO1. PLoS ONE.

[35] Gaikwad A, Long DJ, Stringer JL, Jaiswal AK (2001). In vivo role of NAD(P)H:quinone oxidoreductase 1 (NQO1) in the regulation of intracellular redox state and accumulation of abdominal adipose tissue. J. Biol. Chem..

[36] Ross D, Siegel D (2017). Functions of NQO1 in cellular protection and CoQ(10) metabolism and its potential role as a redox sensitive molecular switch. Front. Physiol..

[37] Hekimi S, Wang Y, Noe A (2016). Mitochondrial ROS and the effectors of the intrinsic apoptotic pathway in aging cells: the discerning killers. Front. Genet..

[38] Reczek CR, Chandel NS (2015). ROS-dependent signal transduction. Curr. Opin. Cell Biol..

[39] Lewis KN (2015). Regulation of Nrf2 signaling and longevity in naturally long-lived rodents. Proc. Natl. Acad. Sci. USA.

[40] Liu XF (2017). Nrf2 as a target for prevention of age-related and diabetic cataracts by against oxidative stress. Aging Cell.

[41] Lee JS (2012). Beta-lapachone, a modulator of NAD metabolism, prevents health declines in aged mice. PLoS One.

[42] Hones J, Muller P, Surridge N (2008). The technology behind glucose meters: Test strips. Diabetes Technol. The.

[43] Goodall CM, Lijinsky W (1976). Oncogenicity tests of p-nitroso-N,N-dimethylaniline and p-nitroso-N,N-diethylaniline in NZR rats and NZO mice. Pathology.

[44] Vatolin SY (1997). Scheduled perturbation in DNA during in vitro differentiation of mouse embryo-derived cells. Mol. Reprod. Dev..

[45] O'Callaghan NJ, Fenech M (2011). A quantitative PCR method for measuring absolute telomere length. Biol. Proced. Online.

[46] Ushakova AV, Grivennikova VG, Ohnishi T, Vinogradov AD (1999). Triton X-100 as a specific inhibitor of the mammalian NADH-ubiquinone oxidoreductase (Complex I). Biochim. Biophys. Acta.

