# Supplementary material for: New drugs for pharmacological extension of replicative life span in normal and progeroid cells
Source: NPJ Aging Mech Dis. 2019 Jan 16;5:2. doi: 10.1038/s41514-018-0032-4 (PMC6335401; doi:10.1038/s41514-018-0032-4)
Supplement: Supplementary file 1 — Supplemental Materials [file 41514_2018_32_MOESM1_ESM.docx]

New drugs for pharmacological extension of replicative life span in normal and progeroid cells

**Authors:**

Sergei Vatolin^1^*, Tomas Radivoyevitch^2^ and Jaroslaw P. Maciejewski^1^

**Affiliations:**

^1^Department of Translational Hematology and Oncology Research, The Cleveland Clinic Foundation

^2^ Department of Quantitative Health Sciences; The Cleveland Clinic Foundation,

*To whom correspondence should be addressed: vatolis2@ccf.org

**Supplementary Materials**

**Chemical reagents.**  violuric acid (Sigma-Aldrich, 95120), N2N1 (Sigma-Aldrich, 114707), Ox12 (MolPort-002-558-027), Ox13 (MolPort-003-800-617, β-lapachone (Apexbio, B2290), dunnione (Cayman chemical, 21943), GssG (Sigma-Aldrich, G4376), insulin (Sigma-Aldrich, I2643), DTNB (Sigma-Aldrich, D218200), cytochrome c (Gold biotechnology, C-640-100) CoQ_10_ (SelectChem, S2398), NADH (Sigma-Aldrich, N8129), NADPH (AlfaAesar, J62089), dicumarole (Sigma-Aldrich, M1390), pHMB (Sigma-Aldrich, 55540), rotenone (Sigma-Aldrich, R8875), antimycin A (Sigma-Aldrich, A8674), and potassium cyanide (Sigma-Aldrich, 60178).

**Antibodies.**

| **Target** | **Host** | **Specificity** | **Clone** | **Dilution** | **Company** | **Catalog#** | **MW, kDa** |
| --- | --- | --- | --- | --- | --- | --- | --- |
| NQO1 | Ms | H | Skiny-1 | 1/300 | AdipoGen | K110525 | 32 |
| Histone H3 | Rb | H, M, R, Mk | D1H2 | 1/1000 | Cell Signaling | 4499S | 17 |
| p21 Waf1/Cip1 | Rb | H, Mk | 12D1 | 1/300 | Cell Signaling | 2947T | 21 |
| p16 | Ms | H |  | 1/200 | BD | 51-1325GR | 16 |
| P-H2A.X | Ms |  | 2F3 | 1/100 | Biolegend | 613408 | 14 |
| Lamin B1 | Rb | H, M, R | D9V6H | 1/500 | Cell Signaling | 13435 | 68 |
| Nrf2 | Rb | H, M, mk | D1Z9C | 1/500 | Cell Signaling | 12721 | 97-100 |

**Enzymatic Assay Conditions for VA (*related to Fig. 3b*).**

Activities were measured under the following conditions:

**Glucose-6-phosphate dehydrogenase (G6PD):** Tris-HCl, pH 8.1, 50mM, MgCl_2_ 1mM, NADP^+^ 0.5mM, glucose-6-phosphate 0.5mM, cell lysate or recombinant protein

**6-phosphogluconate dehydrogenase (6PGD):** Tris-HCl, pH 8.1, 50mM, MgCl_2_ 1mM, NADP^+^ 0.5mM, 6-gluconolactone-phosphate 0.5mM, cell lysate or recombinant protein

**Iso-citrate dehydrogenase (IDH1/2):** Tris-HCl, pH 8.1, 50mM, MgCl_2_ 1mM, NAD(P)^+^ 0.5mM, iso-citrate 0.5mM, cell lysate or recombinant protein

**Thioredoxin reductase (TrxR):** Tris-HCl, pH 7.6 50mM, DTNB 0.5mM, NADPH 1mM, Insulin 0.1mM, cell lysate; ± auranofin 1μM.

**Thioredoxin (Thr):** Potassium phosphate pH 7.2 100mM, DTT 1mM, thioredoxin (recombinant) 2μM, insulin 0.1mM.

**Glutathione reductase (GR):** Potassium phosphate pH 7.5 100mM, glutathione (reduced, GSH) 1mM, NADPH 1mM, diEosin-GssG 1μM, cell lysate or .recombinant protein. ± auranofin 1μM.

Potassium phosphate pH 7.5 100mM, DTMB 1mM, GssG 2mM, NADPH 1mM, cell lysate or recombinant protein. ± auranofin 1μM.

**Peroxiredoxin:** Potassium phosphate pH 7.5 100mM, peroxide 0.1, 0.3, 1 and 3mM, NADPH 1mM, cell lysate or .recombinant protein.

**Pyruvate dehydrogenase (PDH):** TrisHCl, pH7.5 25mM, KCl 50mM, MgCl_2_ 0.2mM, CaCl_2_ 0.01mM, NAD^+^ 1mM, pyruvate 1mM, CoA 0.2mM, cytoplasmic extract. ± lipoic acid (reduced or oxidized) 0.1 mM. ±dichloroacetic acid.

**α-ketoglutarate dehydrogenase (αKGDH):** TrisHCl, pH7.5 25mM, KCl 50mM, MgCl_2_ 0.2mM, CaCl_2_ 0.01mM, NAD^+^ 1mM, α-ketoglutarate 1mM, CoA 0.2mM, cytoplasmic extract.

**Lactate dehydrogenase (LDH):** TrisHCl, pH7.5 25mM, KCl 50mM, MgCl_2_ 0.2mM, CaCl_2_ 0.01mM, NAD^+^ or NADH 1mM, sodium lactate or lactic acid 1mM, cytoplasmic extract.

**Other substrates:** deoxy-glucose, fructose, citrate, malate, glutamate, and glutamine were 1mM

**Table S1.** The absolute values of SAβG (absorbance units at 615 nm) and luciferase activity (luminescence units) that were normalized on control samples and used for preparation of plots on **Fig.1b.** Average and standard deviation values were calculated from 3 independent experiments (3 wells in 384-well plate).

|  |  |  |  |  |  |
| --- | --- | --- | --- | --- | --- |
| **N2N1** |  |  |  | **StDv** |  |
| μM | **Absorbance, 615 nm** | **Luminescence** |  | **Absorbance, 615 nm** | **Luminescence** |
| 0 | 0.226 | 54111 |  | 0.007 | 6101 |
| 0.1 | 0.235 | 56498 |  | 0.006 | 5836 |
| 0.3 | 0.183 | 53050 |  | 0.011 | 2122 |
| 1 | 0.224 | 74801 |  | 0.020 | 1592 |
| 3 | 0.358 | 121485 |  | 0.018 | 8223 |
| 10 | 0.229 | 80901 |  | 0.017 | 3183 |
|  |  |  |  |  |  |
| **VA** |  |  |  | **StDv** |  |
| μM | **Absorbance, 615 nm** | **Luminescence** |  | **Absorbance, 615 nm** | **Luminescence** |
| 0.0 | 0.237 | 52183 |  | 0.007 | 1883 |
| 1.0 | 0.248 | 56788 |  | 0.014 | 1791 |
| 3.0 | 0.253 | 58834 |  | 0.021 | 2302 |
| 10 | 0.217 | 60113 |  | 0.008 | 874 |
| 30 | 0.162 | 82623 |  | 0.038 | 4093 |
| 100 | 0.177 | 81089 |  | 0.013 | 3837 |

**Table S2.** Comet assay did not show any difference between control cells and cells treated with different concentrations of N2N1 or VA. Microphotographs of control, N2N1 (1μM) or VA (30 μM) are shown in **Fig. 2c**.

|  | **μM** | **Object size, μm** | **StDv, ±** | **Tail detection** |
| --- | --- | --- | --- | --- |
| **Control** | 0 | 5.3 | 1.3 | No |
| **N2N1** | 1 | 5.0 | 1.2 | No |
|  | 3 | 5.4 | 1.4 | No |
|  | 10 | 5.9 | 1.5 | No |
| **VA** | 10 | 4.8 | 1.2 | No |
|  | 30 | 4.6 | 1.1 | No |
|  | 100 | 6.0 | 1.5 | No |

**Table S3.** The reaction rates of VA catalyzed reactions with molecular oxygen, hydrogen peroxide and oxidized glutathione as the substrates and NADH as an electron donor. The standard deviation values are shown on the right and calculated from 5 independent experiments (5 wells in 384-well plate).


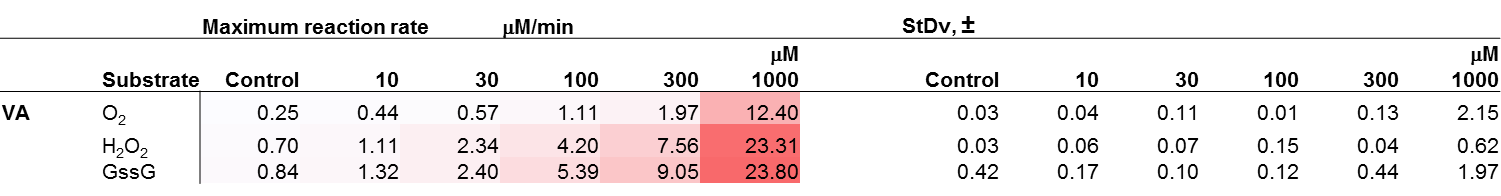

**Table S4.** The rates of N2N1 catalyzed reactions shown on **Fig.4b and c**. The standard deviation values are shown on the right and calculated from 5 independent experiments (5 wells in 384-well plate).

**Table S5.** Reaction rates for N2N1 assisted, NADH-dependent enzymatic reduction of cytochrome c with a set of enzyme specific inhibitors identified NQO1 as a partner protein for N2N1 mediated anti-aging activities. The standard deviation values are shown on the right and calculated from 5 independent experiments (5 wells in 384-well plate).


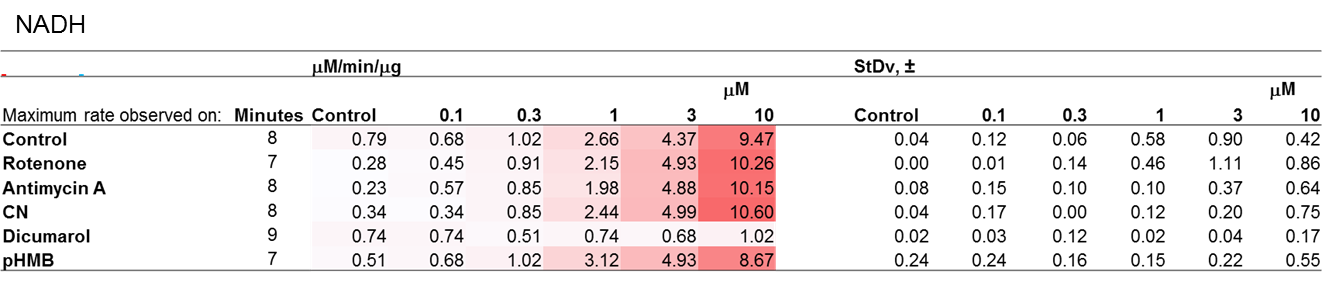


**Table S6.** Reaction rates for N2N1 assisted, NADPH-dependent enzymatic reduction of cytochrome c with a set of enzyme specific inhibitors confirmed that NQO1 is a partner protein for N2N1 mediated anti-aging activities. The standard deviation values are shown on the right and calculated from 5 independent experiments (5 wells in 384-well plate).


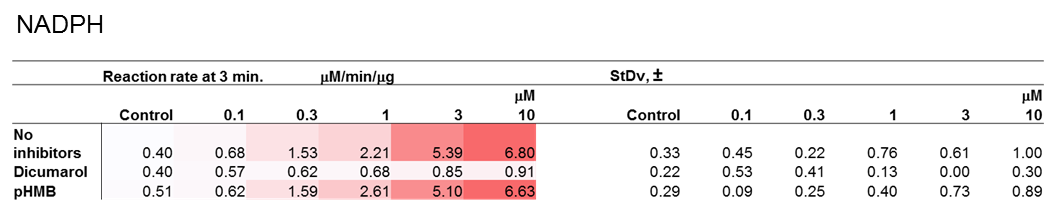


**Table S7.** The rates of N2N1 driven reduction of cytochrome c or CoQ10 in the presence of inhibitors. The standard deviation values are shown on the right. **(a)** The rates of CoQ10 reduction measured as decrease of absorbance at 340 nm (NADH oxidation). **(b)** The rates of cytochrome c reduction assisted by 1mM of N2N1 in cytoplasmic extracts prepared from old fibroblasts (55PD), proliferating young fibroblasts (33PD) or young fibroblast (33PD) kept in monolayer for one week. The standard deviation values are shown on the right and calculated from 5 independent experiments (5 wells in 384-well plate).

**(a)**


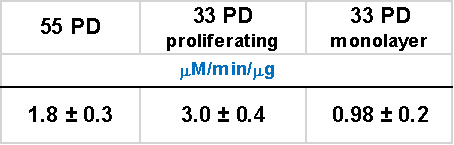
**(b)**

**
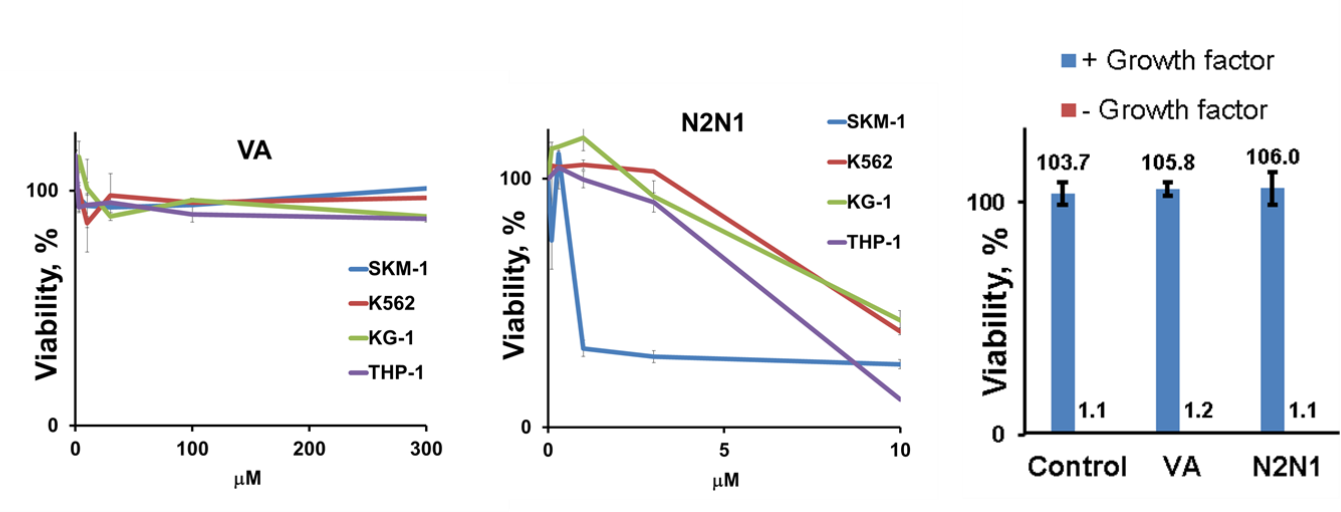
**

**Fig. S1.** **(a)** Treatment with VA of cancer cell lines was neither toxic nor pro-proliferative. N2N1 was partially toxic for the tested cell lines (middle panel). **(b)** VA and N2N1 do not support the growth of erythropoietin-dependent UT7 cells. The standard deviation value is shown as an error bar and it was calculated from 3 independent experiments.

**
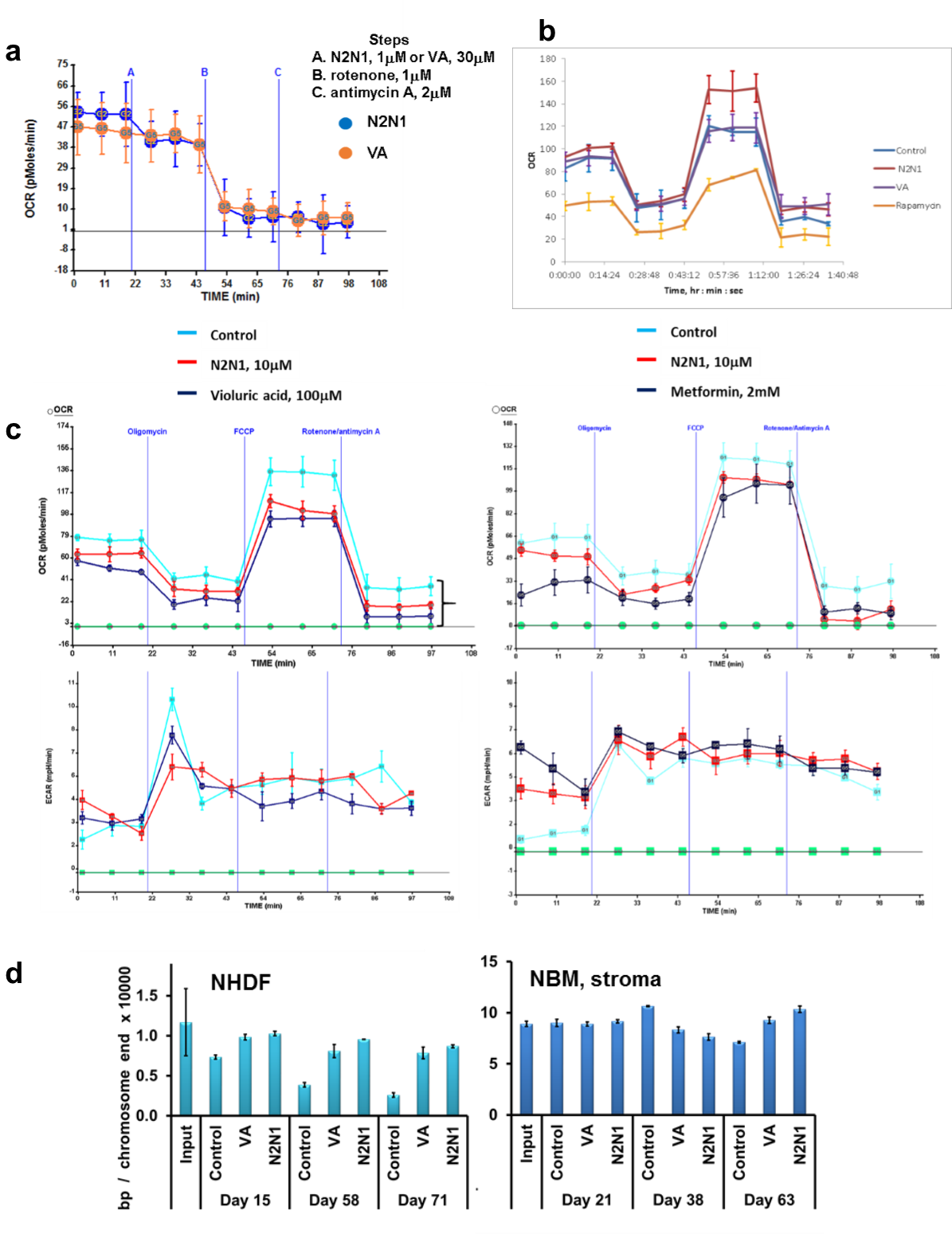
**

**Fig. S2.** Neither the mitochondrial respiration / glycolysis nor telomere length were affected by treatment with N2N1 or VA. The standard deviation value is shown as an error bar and it was calculated from 3 independent experiments. **(a)** The result of N2N1 (1 μM) or VA (30 μM) treatment was assessed right after addition of the drugs to the overnight culture of NHDF followed by addition of oxidative phosphorylation inhibitors. **(b)** NHDF were pre-incubated with N2N1 (1 μM) or VA (30 μM) for 10 days and mitochondrial respiration was assessed using standard oxidative phosphorylation inhibitors. Rapamycin treatment (1nM) was used as a positive, anti-aging control. Mitochondrial respiration rate (OCR) is shown. N2N1 treatment may increase so-called spare respiratory capacity. **(c)** Cells (NHDF) were pre-incubated with increased concentrations of N2N1 (10 μM) or VA (100 μM) for 24 hours followed by addition of standard array of oxidative phosphorylation inhibitors. Metformin is shown as a possible control. Molecular oxygen consumption rate (OCR) is shown as a measure of mitochondrial respiration. Extracellular acidification rate (ECAR) was used to measure glycolytic activity. No statistically significant differences between control and treatments were observed. **(d)** The telomere length changed in NHDF after treatment with N2N1 (1 μM) or VA (30 μM). Stromal cells entered senescence crisis with long telomeres and treatment with VA or N2N1 extended RLS, but did not change the telomere length in this cell type. The standard deviation value is shown as an error bar and it was calculated from 3 independent experiments.


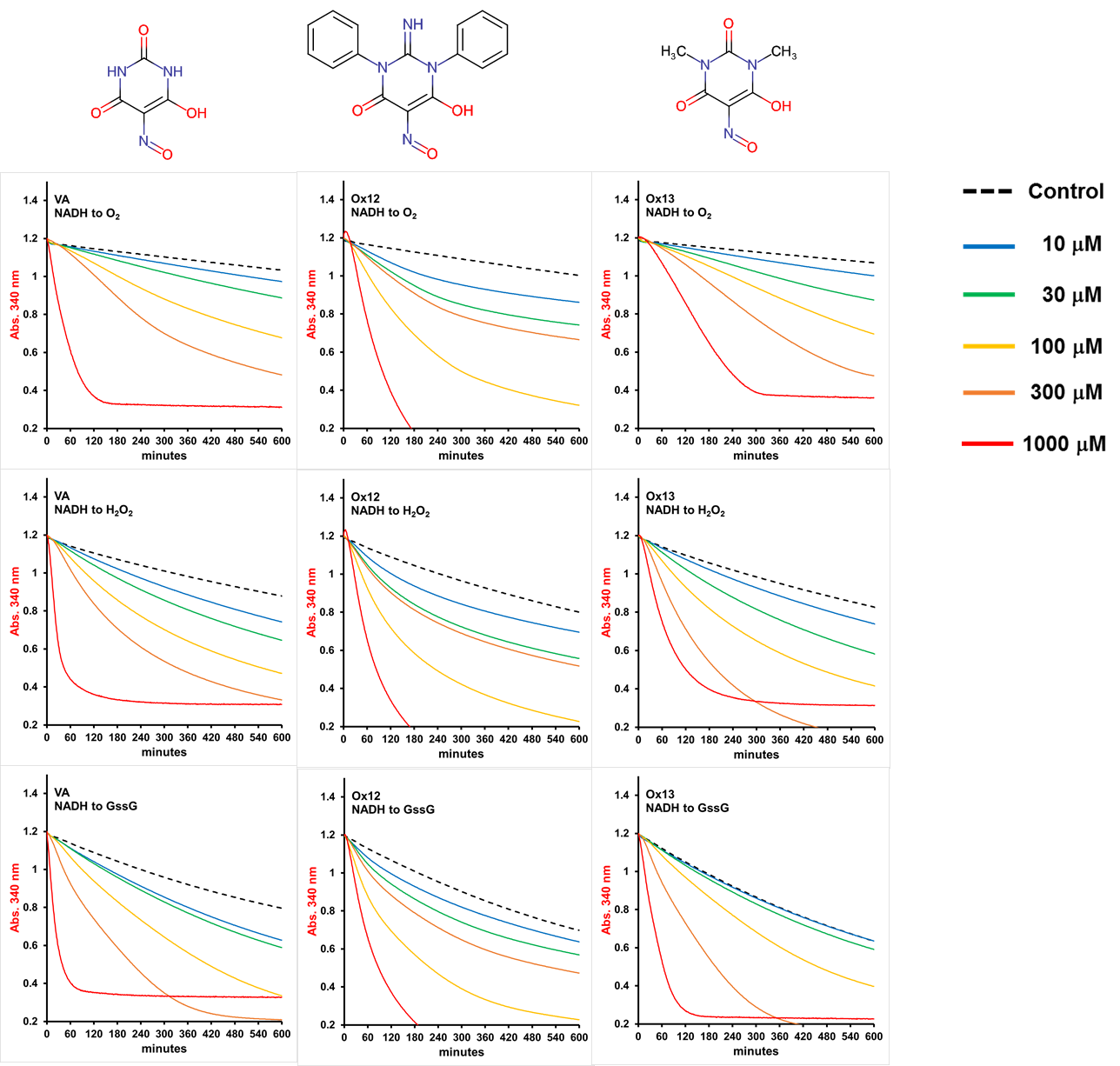


**a**

**b**

**c**

**d**

**e**


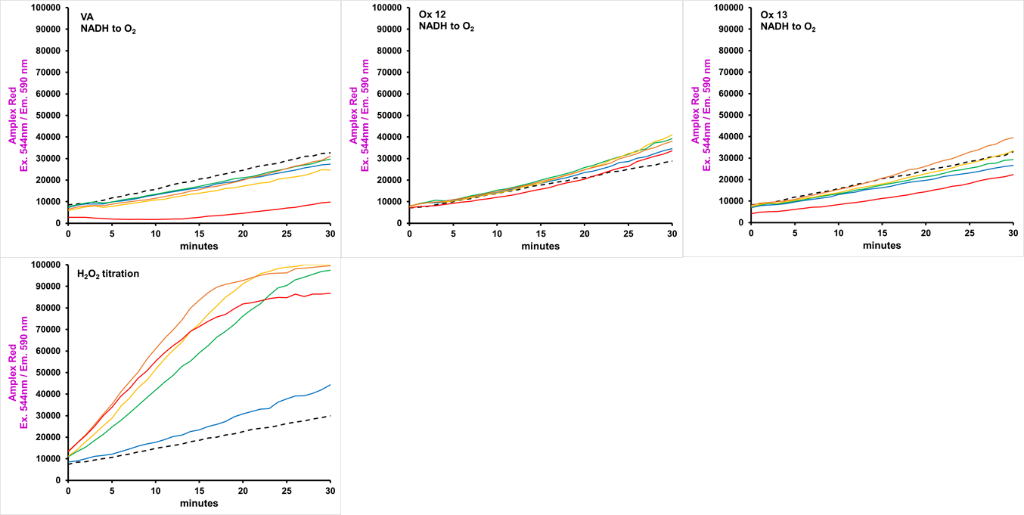
**f**

**Fig. S3.** Identification and validation mechanism of action of VA and its derivatives Ox12 and Ox13. **(a)** Molecular structures of VA and its derivatives Ox12 and Ox13. **(b)** VA and its modified analogs Ox12 and Ox13 at high concentration can oxidize NADH indicating possible transfer electrons to molecular oxygen naturally dissolved in reaction buffer. Addition of phenyl groups (Ox12) resulted in better reaction rate, while addition of methyl groups (Ox13) decreased reaction rate. **(c)** Addition of hydrogen peroxide (5 mM) to the reaction mix increase the reaction rates of VA and Ox13 catalyzed reactions, but not Ox12’s. **(d)** Addition of oxidized glutathione (GssG, 0.5mM) to the reaction mix increase the reaction rates of VA and Ox13 catalyzed reactions, but not Ox12’s. **(e)** The oxidation of NADH were monitored as an absorbance decrease at 340 nm. The reaction rates (in μM/min/μg) are shown in table. The standard deviation values are shown on the right and calculated from 5 independent experiments (5 wells in 384-well plate). **(f)** None of the tested compounds caused the generation of ROS that was evaluated by Amplex red assay. The H_2_O_2_ titration curves are shown.

**
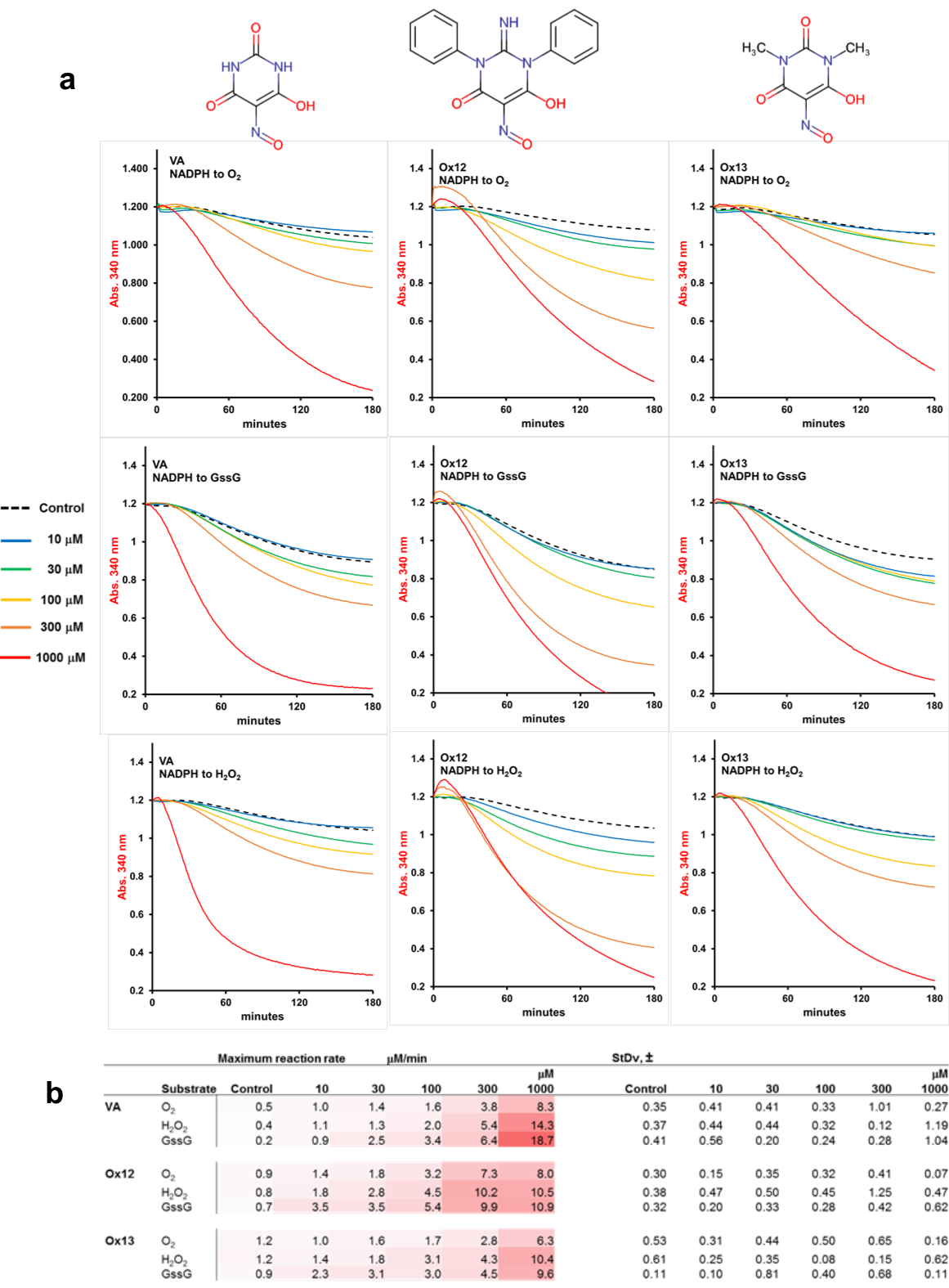
**

**Fig. S4. (a)** NADPH can also support the VA mediated electron transfer to corresponding substrates. O2, hydrogen peroxide and oxidized glutathione were tested with VA, Ox12 and Ox13.

**(b)** The oxidation of NADPH were monitored as an absorbance decrease at 340 nm. Reaction rates (in μM/min/μg) were as shown. The standard deviation values are shown on the right and calculated from 5 independent experiments (5 wells in 384-well plate).


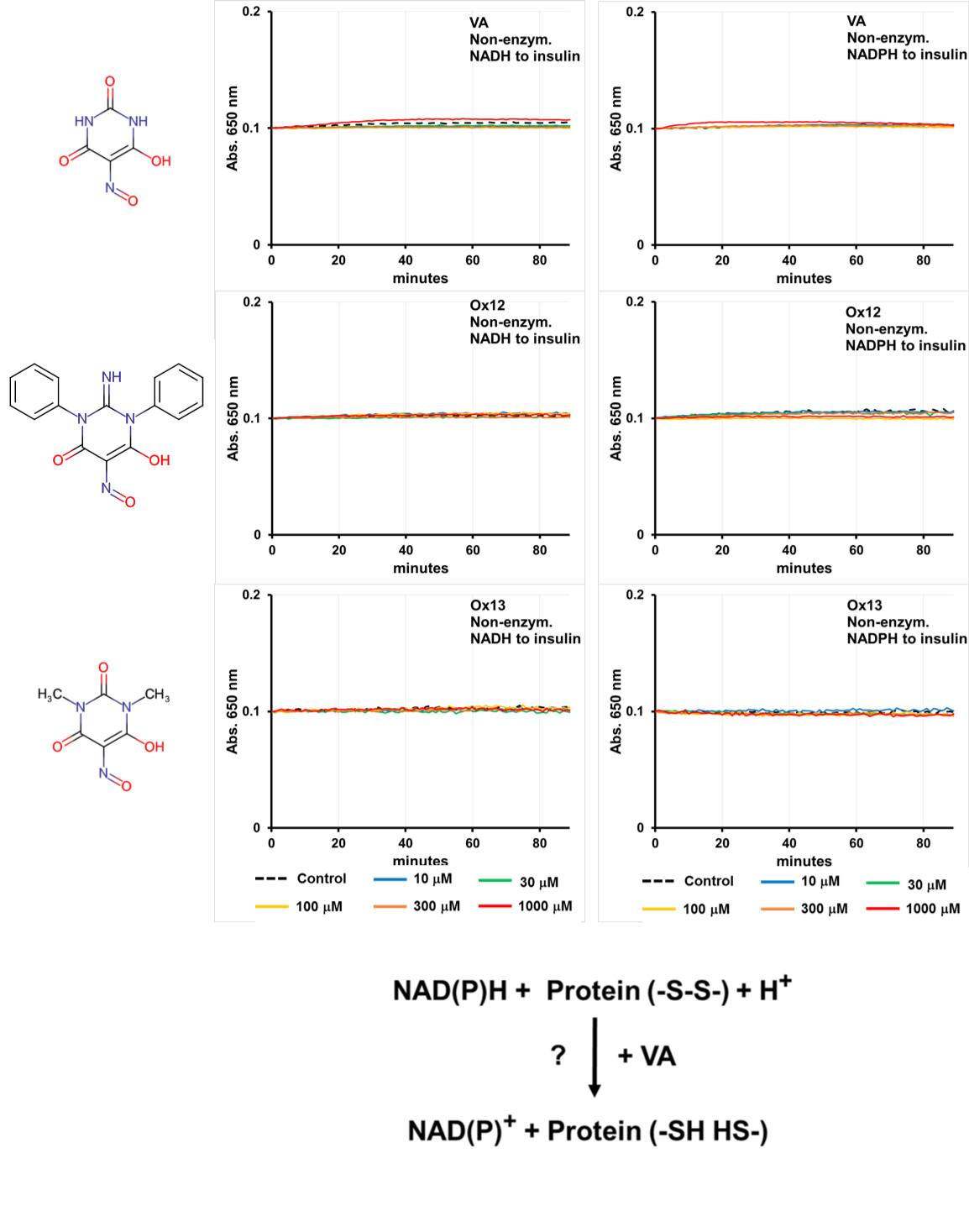


**Fig. S5.** VA does not catalyze the reduction of disulfide bonds inside a protein or between the proteins. VA and its derivatives, Ox12 and Ox13 were incubated with either NADH or NADPH and insulin as an electron acceptor onto its intramolecular disulfide bonds. The reduction of cysteines would have resulted in decreased solubility of insulin and corresponding increase in absorbance at 650 nm.


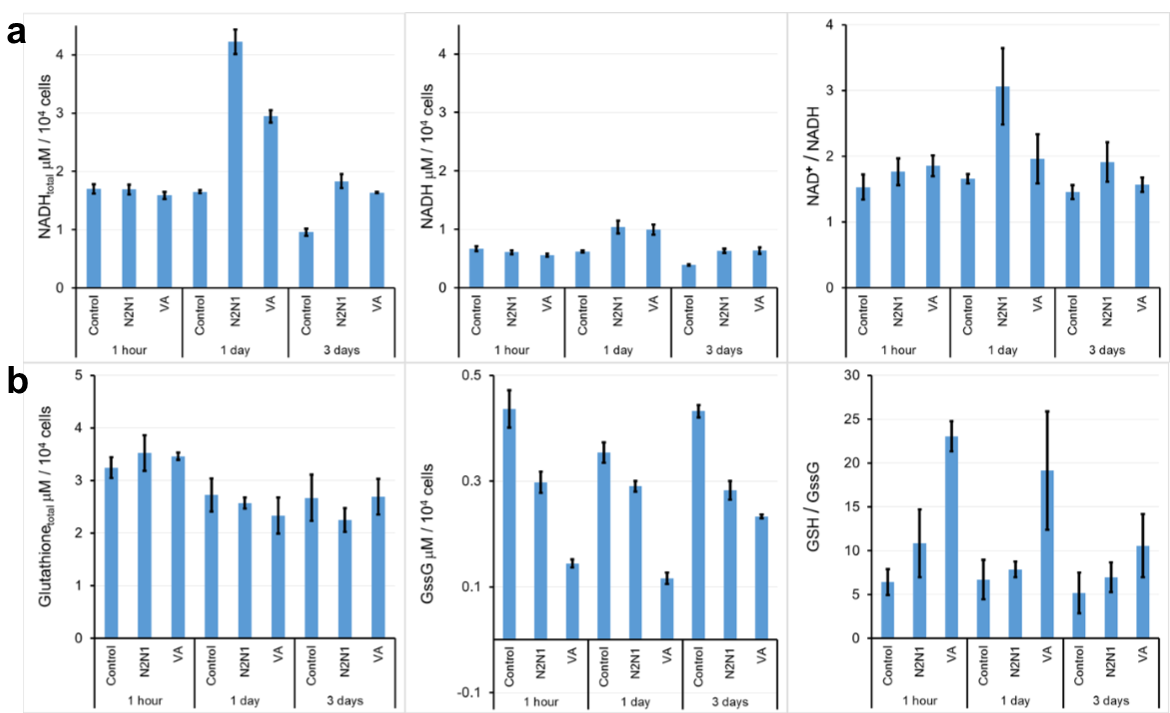


**Fig. S6.** The evaluation of GSH/GssG and NAD^+^/NADH ratios in NHDF (37 PD) after treatment with N2N1 (1 μM) or VA (30 μM) for one hour and for one or three days. **(a)** Treatment with N2N1 resulted in increase of NAD^+^/NADH ratio after one day of incubation, **(b)** while VA managed to increase the target GSH/GssG ratio just after one hour. The elevated level was observed at least for three days. The standard deviation value is shown as an error bar and it was calculated from 3 independent experiments.


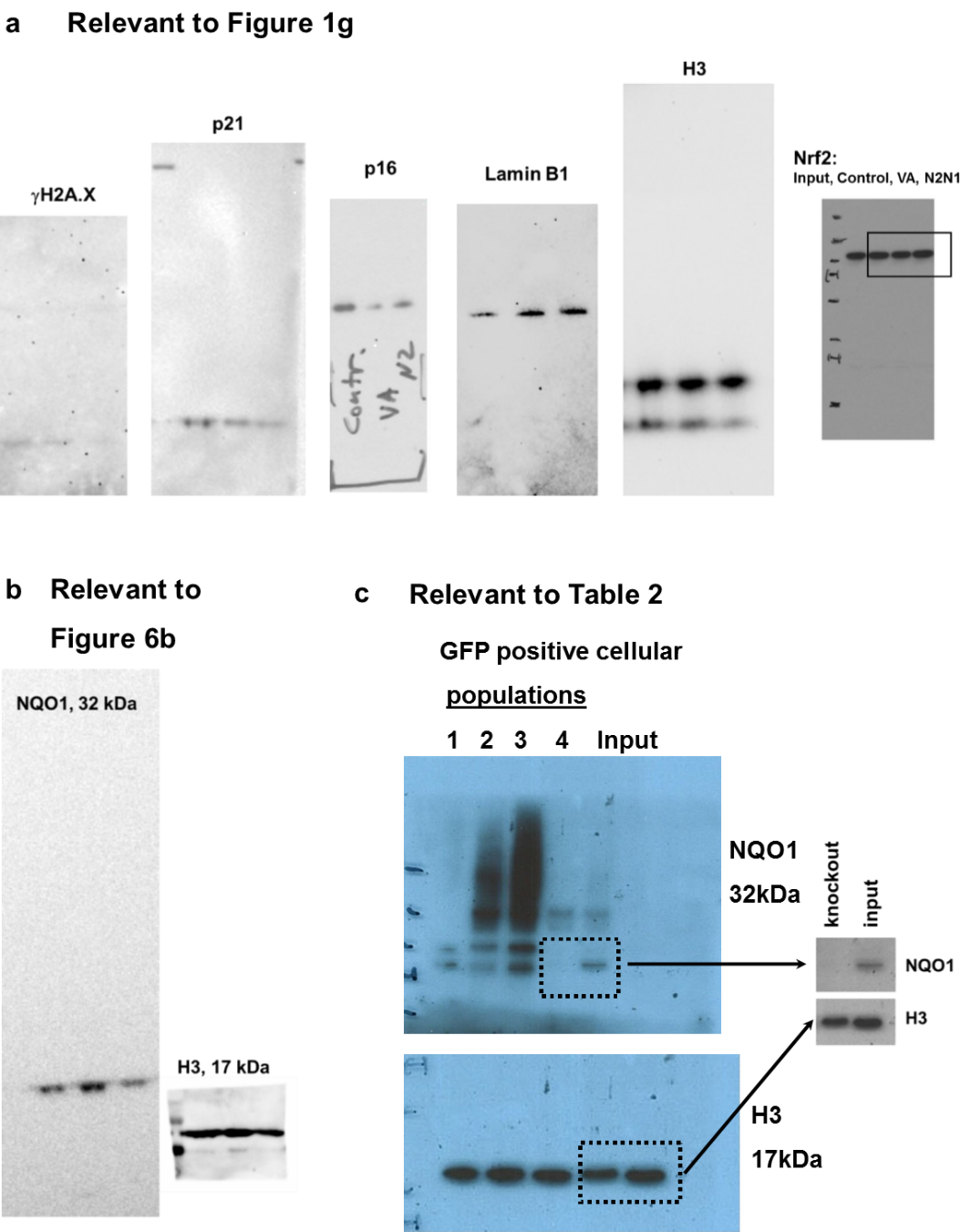


**Fig. S7.** The original images of shown western blots used in: **(a)** Fig. 1g; **(b)** in Fig 6b.

**(c)** After transfection of CRISPR/Cas9 plasmid into NHDF, 5 GFP positive cells were collected into a single well of 96-well plate using a cell sorter. Total 4 cellular population were established (out of 12) and NQO1 protein level was analyzed by western blot. Three samples did not show NQO1 negative phenotype, one cell population was suitable for further experiments with RLS extending drugs. Input level of NQO1 (cells before transfection) is shown on the right lane. Histone H3 was used as a loading control.
